# Magnetic-responsive hydrogels as composite smart materials: preparation, applications and challenges

**DOI:** 10.1039/d6ra08388a

**Published:** 2026-11-02

**Authors:** Meng Zhang, Xin Cui, Yanbin Zhang, Jian Zhang, Zongxu Chen, Xuesong Chang, Xing Zhao, Mingzheng Liu, Changhe Li

**Affiliations:** a School of Mechanical and Automotive Engineering, Qingdao University of Technology Qingdao 266520 China cuixin@qut.edu.cn; b College of Aerospace and Mechanical, Shandong University of Aeronautics Binzhou 256600 China; c Quanzhou University of Information Engineering Quanzhou 362000 China; d Hisense Refrigerator Co., Ltd Qingdao 266700 China

## Abstract

Magnetic-responsive hydrogels (MRHs) combine hydrogel networks with magnetic particles, enabling remote actuation, magnetothermal conversion, and multifunctional responses. However, their performance is highly dependent on particle composition, size, loading, dispersion, particle–polymer interactions, crosslinking density, and fabrication conditions, making their design and characterization complex. Moreover, systematic relationships among preparation, interfacial modification, structure–property regulation, and application performance remain insufficiently understood. To address these issues, this review summarizes the preparation, modification, properties, and applications of MRHs from a preparation–interface–property–application perspective. Firstly, soft- and hard-magnetic hydrogels are classified and compared. Next, blending, *in situ* precipitation, and covalent bonding are discussed with emphasis on particle dispersion, retention, interfacial stability, and mechanical performance. Furthermore, surface modification, mechanical reinforcement, and the effects of particle characteristics, loading, and crosslinking density on magnetic, swelling, mechanical, magnetothermal, and biological properties are analyzed. Finally, applications in biomedicine, environmental remediation, actuation and sensing, and emerging engineering fields are reviewed together with challenges in nanoparticle leakage, fatigue, magnetic-field dosimetry, biodegradation, reproducibility, sterilization, and scale-up. Overall, this review provides guidance for the rational design and practical development of MRHs.

## Abstract 2

This review evaluates the advantages and limitations of preparation methods for magnetic-responsive hydrogels, examines the effects of modifiers and key parameters on their properties, and discusses application challenges and improvements.

## Graphical abstract

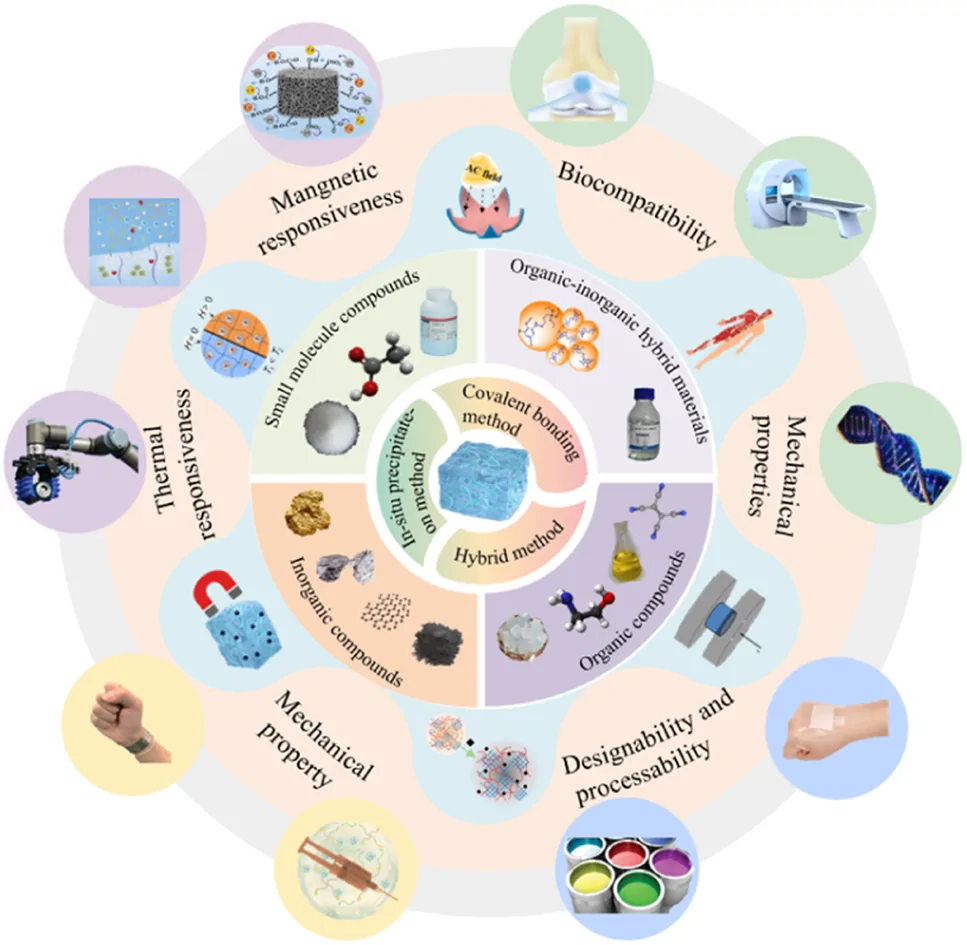

## 1 Introduction

Hydrogels are hydrophilic polymer materials with a three-dimensional network structure. Owing to their characteristic “wet, soft, and elastic” properties and similarity to biological tissues, they have been widely studied in biomedicine, tissue engineering, and flexible electronics.^1–5^ However, traditional hydrogels are relatively static and functionally limited. As materials science advances toward intelligent and functional systems, increasing demands for dynamic, controllable, and precise interactions have driven the development of smart responsive hydrogels. These materials detect environmental changes, such as temperature, pH, light, electric fields, or magnetic fields, and convert them into reversible changes in physical states (*e.g.*, shape, volume, and permeability) or chemical properties.^6–8^ Among these stimuli, magnetic fields are particularly attractive because they enable non-contact and remotely controlled actuation with high spatiotemporal controllability. Unlike optical stimulation, magnetic fields do not require direct optical access to the responsive material; however, the effective force, torque, or heating generated in MRHs depends strongly on magnetic-field strength and gradient, source-to-target distance, particle magnetization and concentration, and the geometry of the target environment. These characteristics make magnetic stimulation particularly useful for remotely manipulating responsive materials in complex biological systems and confined spaces.^9^ Consequently, magnetic-responsive hydrogels (MRHs), formed by integrating magnetic particles into hydrogel matrices, show substantial potential across diverse applications.

As shown in Fig. 1, extensive theoretical and experimental studies have been conducted on MRHs worldwide. Early research focused mainly on fundamental principles, the transition from conventional hydrogels to MRHs, and preliminary investigations of their properties and applications. To expand their applications, researchers subsequently explored strategies such as double-network construction, nano-clay incorporation, and magnetic-field induction to improve MRHs performance, particularly mechanical strength and leakage resistance. More recently, research has increasingly focused on extending the functionality of MRHs across diverse application scenarios.

**Fig. 1:**
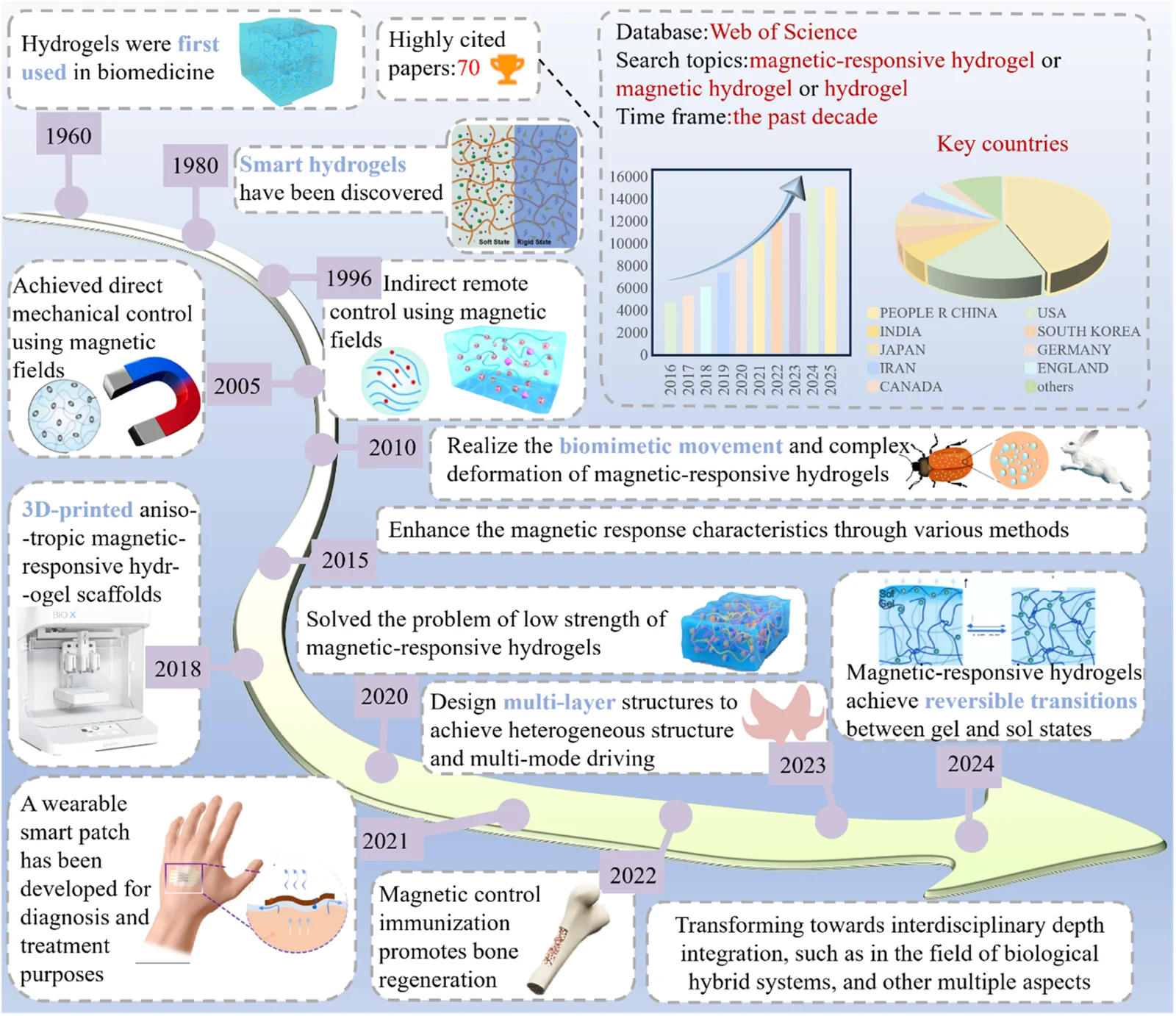
Development history of magnetic-responsive hydrogels.

However, MRH preparation methods are diverse and technically complex, making their performance difficult to control. The wide range of enhancement strategies further complicates method selection, thereby limiting broader application. As shown in Table 1, several reviews have addressed specific aspects of magnetic-responsive hydrogels, including tissue engineering, biomedical applications, flexible magnetic actuators, and cartilage regeneration. More recent reviews have further summarized their fabrication and biomedical applications. However, comparatively less attention has been paid to establishing an integrated relationship among magnetic-particle characteristics, incorporation strategies, interfacial modification, mechanical reinforcement, and application-specific performance. In particular, systematic comparisons of nanoparticle dispersion and leakage, particle–polymer interactions, preparation–property relationships, and the applicability of MRHs beyond biomedical scenarios remain limited.

**Table 1:** Comparison of representative reviews on magnetic-responsive hydrogels and the scope of the present review

Review	Main focus	Major topics covered	Compared present review
Liu *et al.*, 2020 ^10^	Tissue engineering	Preparation; bone, cartilage and neural tissue engineering	Limited coverage of surface modification and multiple application fields
Li *et al.*, 2021 ^9^	Biomedical applications	Preparation, magnetic response mode, medical application	Limited analysis in surface modification and multiple application fields
Grimaudo *et al.*, 2022^11^	Neural tissue response	Bioactive injectable hydrogels	Lack of preparation, properties, and multiple applications
He *et al.*, 2023 ^12^	Flexible magnetic hydrogel actuators	Actuator design, magnetization, mechanical reinforcement, soft robots	Strongly actuator-oriented
Taghizadeh *et al.*, 2024 ^13^	Articular cartilage	Magnetic hydrogels for cartilage tissue engineering	Application-specific
Suhail *et al.*, 2025 ^14^	Biomedical field	Preparation, classification, biomedical applications	Limited description of multiple applications and surface modifications
Londhe *et al.*, 2025 ^15^	Biomedical engineering	Fabrication, properties, biomedical applications	Mainly biomedical engineering
Present review	Preparation, surface modification, properties and applications	Soft/hard magnetic systems; blending/*in situ*/covalent routes; MNP surface modification; mechanical enhancement; biomedical, environmental, actuation/sensing and emerging applications	Emphasizes cross-comparison, structure–property relationships and translational barriers

Key scientific challenges in MRHs research include characterizing their properties and improving their mechanical performance. However, characterization of MRHs is particularly challenging because their magnetic, mechanical, swelling, and thermal behaviors are strongly coupled. The macroscopic properties of magnetic hydrogels are affected by magnetic-particle composition, concentration, size, distribution, particle–polymer interactions, network crosslinking degree, swelling behavior, and preparation method.^16,17^ For example, increasing magnetic-particle loading may enhance saturation magnetization and magnetothermal conversion, but may simultaneously promote particle aggregation, alter crosslinking density, and change the stiffness and swelling behavior of the hydrogel. Similarly, particle size and surface functionalization affect not only magnetic properties but also interfacial bonding, dispersion, particle leakage, and cytocompatibility.^17,18^ Crosslinking density further regulates network mesh size, water uptake, nanoparticle mobility, and mechanical properties.^19^ Therefore, reliable evaluation of MRHs requires complementary characterization techniques, including magnetic hysteresis measurements, rheological and mechanical testing, swelling analysis, microscopic characterization of particle distribution, particle-leaching measurements, and temperature evolution under alternating magnetic fields.^18^ In addition, magnetic-field parameters such as field strength, gradient, frequency, exposure duration, and sample-to-magnet distance should be reported because the measured magnetic response is strongly dependent on the testing configuration. For meaningful comparison among different MRH systems, magnetic parameters such as saturation magnetization (*M*_s_), remanent magnetization (*M*_r_), and coercivity (*H*_c_) should be measured using vibrating-sample magnetometry or SQUID magnetometry and reported together with the normalization basis of magnetization.^18^ Mechanical properties should be evaluated under defined hydration state, strain rate, and loading mode, whereas swelling measurements should specify medium composition, pH, temperature, and equilibrium time.^19^ For magnetothermal measurements, the magnetic-field amplitude, frequency, exposure duration, nanoparticle concentration, sample volume, and initial temperature should be reported because differences in field conditions, apparatus, sample geometry, and analysis method can substantially affect the resulting heating metrics.^20,21^ Particle distribution and leakage may be evaluated using microscopy together with elemental or mass-balance analyses. Without such experimental context, numerical values obtained from different MRH systems are not directly comparable.

Accordingly, this review systematically summarizes recent advances in MRHs, as outlined in Fig. 2. It compares major preparation methods and their advantages and limitations, examines materials used to modify magnetic nanoparticles and their effects, summarizes the properties of MRHs properties and mechanical enhancement strategies, reviews major application areas, and identifies future research directions. The aim is to provide theoretical and technical guidance for improving MRH performance and expanding their applications.

**Fig. 2:**
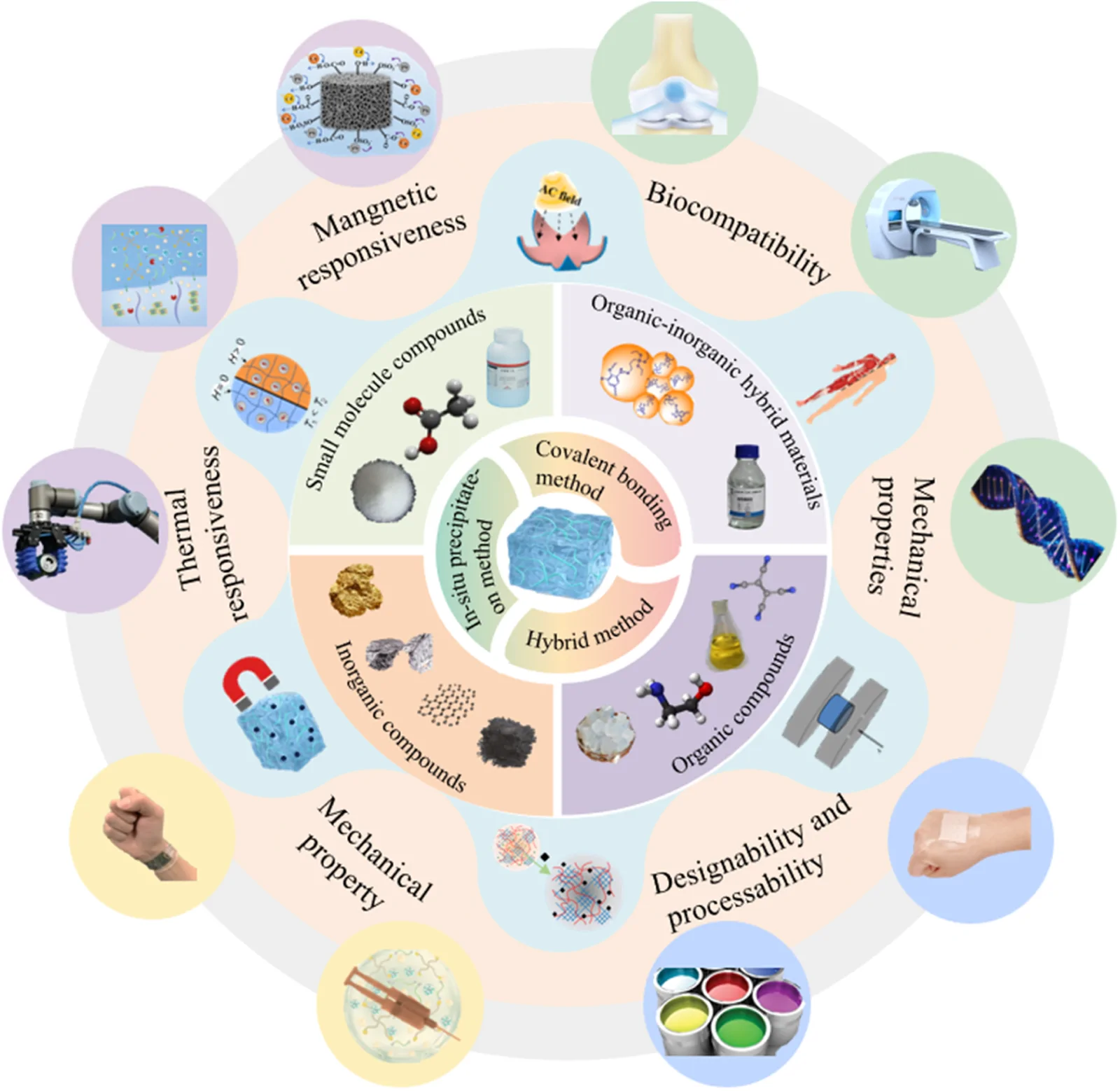
Overall structure of the paper.

## 2 Preparation methods of different kinds of magnetic-responsive hydrogels

MRHs are smart hydrogels that respond to external magnetic fields by incorporating magnetic particles into a hydrogel polymer network. Based on the intrinsic properties of these particles, MRHs are generally classified as soft-magnetic or hard-magnetic hydrogels. Their fabrication mainly involves three approaches: physical mixing, *in situ* precipitation, and covalent bonding.

### 2.1 Classification and basic properties of hydrogels

Hydrogels are three-dimensional hydrophilic polymer networks composed of natural or synthetic polymers. As shown in Fig. 3, they can be classified by polymer source, crosslinking type, environmental responsiveness, and particle size.^22,23^

**Fig. 3:**
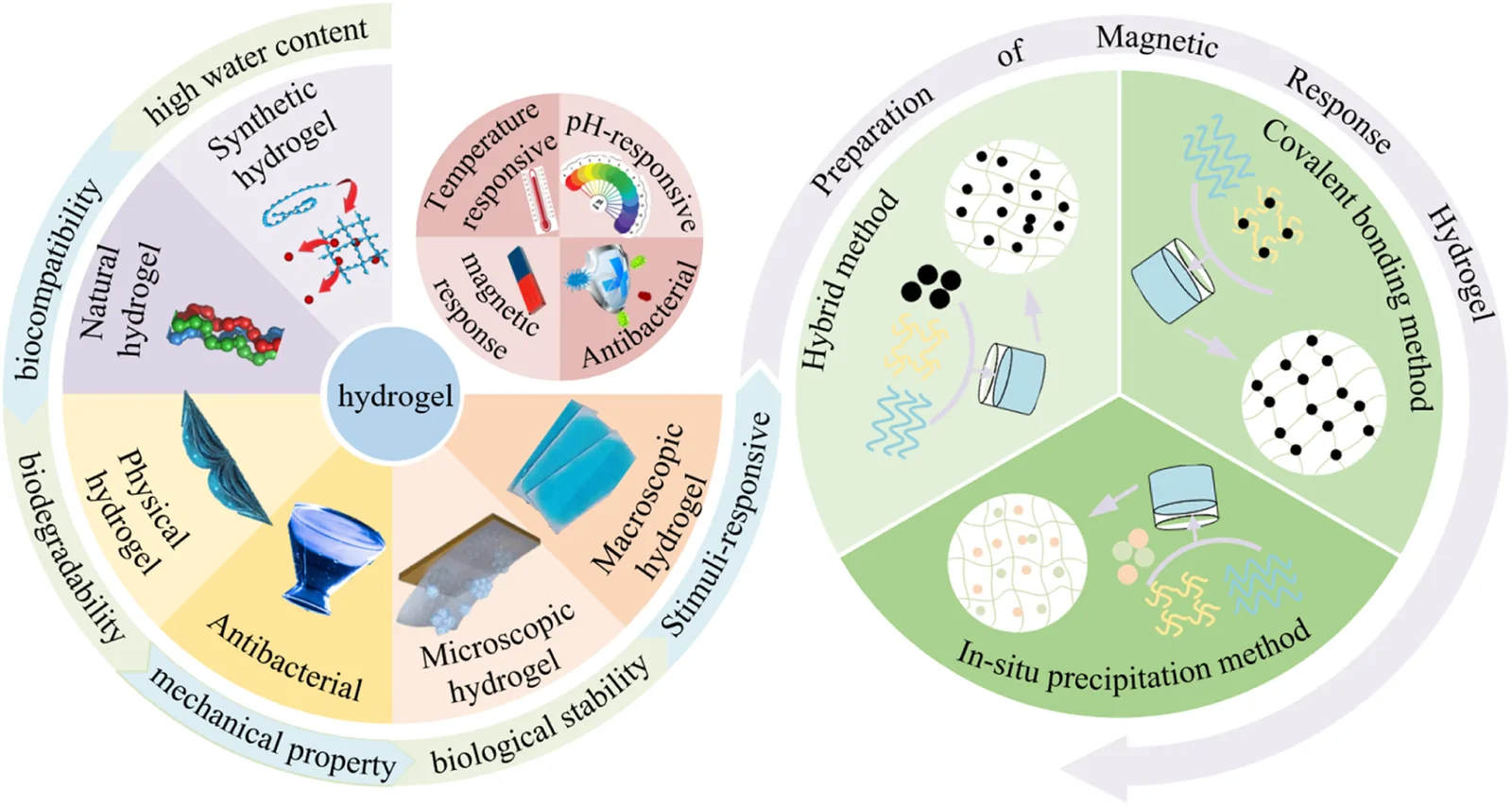
Overview of hydrogels.

#### 2.1.1 Classification based on source

Polymer source is a key criterion for hydrogel classification. Accordingly, hydrogels are broadly divided into natural and synthetic types.

Natural hydrogels are formed from naturally derived polymers such as gelatin, collagen, chitosan, silk fibroin, hyaluronic acid, cellulose, and alginate.^24,25^ Depending on their source, purification, crosslinking chemistry, and degradation products, these materials often exhibit favorable cytocompatibility and biodegradability, enabling gradual degradation in biological systems and reducing risks associated with long-term tissue retention. They are therefore widely used in food and biomedical applications. For example, gelatin-based hydrogels are commonly used as wound dressings for hemostatic, antibacterial, and anti-inflammatory purposes.^26^

Synthetic hydrogels are constructed from synthetic polymers or monomers, including hydrophilic materials such as polyethylene glycol (PEG), polyvinyl alcohol (PVA), polyacrylamide (PAM), and poly(acrylic acid) (PAA), and can be formed through either chemical or physical crosslinking.^22,27,28^ They generally exhibit higher mechanical strength and greater chemical tunability, with physicochemical properties adjustable through monomer and crosslinker ratios. These hydrogels are widely used in tissue engineering, controlled drug delivery, and artificial organs. For example, PVA hydrogels combine good biocompatibility, mechanical strength, and low friction, while their crosslinking density can be tuned by monomer concentration. They have been applied in tissue engineering scaffolds, articular cartilage, and electronic skin.^28^

#### 2.1.2 Classification based on crosslinking method

Hydrogels can be classified by crosslinking type into chemically and physically crosslinked hydrogels.^29,30^

Chemically crosslinked hydrogels form stable three-dimensional networks through covalent bonds and are generally irreversible unless these bonds are broken. Common methods include free-radical polymerization and condensation reactions. Their stable networks provide high mechanical strength and resistance to deformation under complex physiological conditions, making them suitable for tissue engineering scaffolds and artificial joints. For example, chemically crosslinked polyvinyl alcohol (PVA) hydrogels can exhibit enhanced mechanical properties, electrical conductivity, and fatigue resistance, supporting applications in sensors and flexible electronics.^31^

Physically crosslinked hydrogels form networks through non-covalent interactions, including ionic interactions, hydrogen bonding, and crystallization. These networks typically form under mild conditions and are reversible, providing high swelling capacity and structural adaptability to external stimuli. They are therefore suitable for dynamic applications such as controlled drug delivery and soft robotics. For example, hydrogels formed by polymerizing and crosslinking 3-2-(methylacryloyloxy)ethyl-1-propanesulfonate (SBMA) monomers are used as postoperative barriers because of their resistance to protein adsorption and fibroblast adhesion.^32^

#### 2.1.3 Classification based on environmental responsiveness

Based on environmental responsiveness, hydrogels are classified as conventional (non-smart) or smart (environmentally sensitive) hydrogels.

Conventional hydrogels have simple structures, straightforward fabrication, and relatively low production costs. They are widely used in biomedicine and the food industry, for example, as tissue repair materials and food preservatives.^33^ Their stability and low cost are advantageous for large-scale applications with limited performance requirements. However, their relatively fixed properties and lack of environmental responsiveness restrict their use in advanced applications.

Smart hydrogels can sense external stimuli and undergo corresponding changes in their physical structure or chemical properties, often enabling controlled absorption and release.^34–40^ Major types include photo-responsive, thermo-responsive, pH-responsive, and magnetic-responsive hydrogels.^41–47^ Thermo-responsive hydrogels swell or shrink within specific temperature ranges and can regulate drug release through temperature changes;^48–50^ pH-responsive hydrogels alter their structure and properties with environmental pH and are used in biosensing and targeted drug delivery. Tissue-regenerative hydrogels promote wound healing and regeneration through local delivery to injury sites;^51–54^ whereas antibacterial hydrogels can deliver antibiotics to infected wounds with controllable release profiles.^29,55^ Among smart hydrogels, MRHs exhibit rapid magnetic responsiveness, spatiotemporal control, and non-invasive remote actuation. Applied magnetic fields can remotely induce deformation, movement, and other behaviors.^56^ As shown in Fig. 4, MRHs have been applied in remote drug/cell delivery, magnetic hyperthermia, tissue engineering, magnetic resonance imaging, and soft actuators.^44,57–73^ More broadly, the environmental responsiveness of smart hydrogels supports applications in biomedicine, environmental monitoring, and smart materials.^69,73–75^

**Fig. 4:**
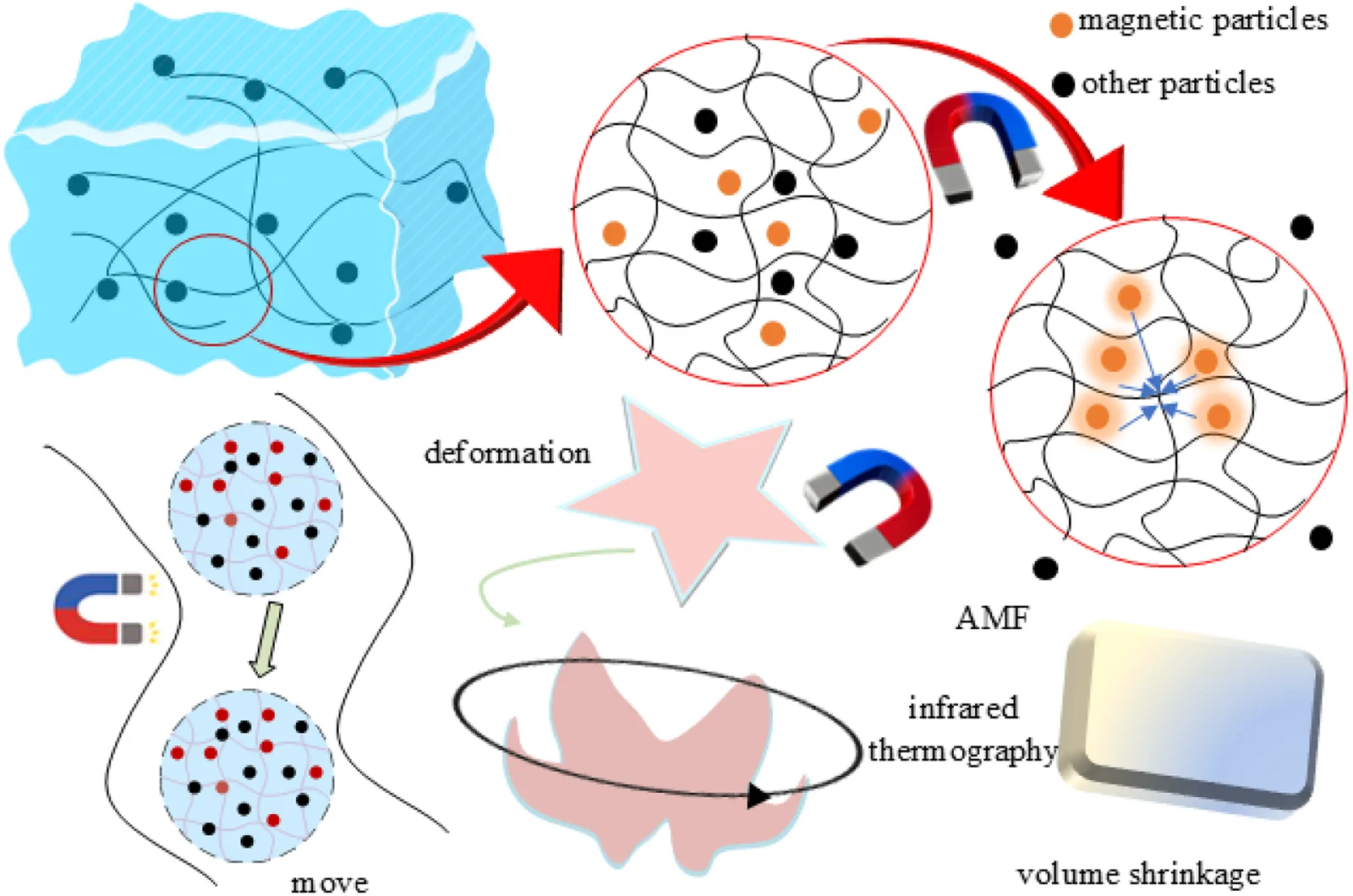
Schematic of magnetic-responsive hydrogel responses.

#### 2.1.4 Classification based on size

Based on particle size, hydrogels can be classified as microscopic or macroscopic hydrogels.

Microscopic hydrogels typically range from nanometers to micrometers. Their small size and high specific surface area provide distinctive physicochemical properties, enabling applications in drug delivery, enzyme immobilization, catalysis, and pollutant adsorption. For example, nanoscale microgels can serve as injectable drug carriers for site-specific release, improving therapeutic efficacy while reducing side effects. Microgels can also adsorb heavy metal ions from wastewater. In addition, nanocomposite hydrogels incorporate nanomaterials such as nanoparticles, graphene, or carbon nanotubes to enhance mechanical, electrical, or magnetic properties.

Macroscopic hydrogels generally range from millimeters to centimeters and can be fabricated as rods, spheres, porous sponges, matrices, films, or fibers. Their large size facilitates handling and supports applications such as agricultural water retention, packaging films, and sealing materials. They are typically produced by bulk polymerization and exhibit sufficient mechanical stability for practical use. For example, thick hydrogel sheets can function as gaskets to reduce liquid leakage and evaporation.

#### 2.1.5 Basic properties of hydrogels

Hydrogels exhibit high water content, biocompatibility, biodegradability, mechanical performance, stimulus responsiveness, and biological stability.^22,76–78^ Their water content can exceed 90% of their mass, providing softness, lubrication, and structural stability in moist environments.^23,79,80^ Many hydrogels also exhibit good biocompatibility, enzymatic stability, and antioxidant activity, enabling prolonged residence in biological systems with limited immune or toxic responses.^68,81^ Their stimulus responsiveness further allows them to adapt to environmental changes, supporting applications in biomedicine, agriculture, and industry.^77,82–87^

### 2.2 Classification of magnetic-responsive hydrogels

As smart hydrogels, MRHs can undergo magnetic-field-induced deformation, including elongation, contraction, and bending, without disrupting their network structure. MRHs can be broadly classified as soft- or hard-magnetic systems according primarily to the hysteresis behavior of the incorporated magnetic phase, particularly its coercivity and remanent magnetization, as shown in Table 2. Particle composition alone is insufficient for this classification because magnetic behavior also depends strongly on particle size, crystallinity, morphology, and domain state.

**Table 2:** Classification and comparison of magnetic-responsive hydrogels

Parameter category	Soft magnetic hydrogel	Hard magnetic hydrogel
Magnetic particle	Fe_3_O_4_, γ-Fe_2_O_3_, ultrasmall CoFe_2_O_4_	Co, Nd_2_Fe_14_B, CoFe_2_O_4_
Particle size	5–30 nm (Fe_3_O_4_-based, 0.25–15 wt%)^60,88,89^	20–100 nm (CoFe_2_O_4_); 5–50 µm (Nd_2_Fe_14_B)^60,88,89^
Saturation magnetization (*M*_s_)	3–20 emu g^−1^ (Fe_3_O_4_-rich, 0.25–15 wt%)^60,63,89^	10–180 emu g^−1^ (CoFe_2_O_4_/Nd_2_Fe_14_B, >5 wt% or 7.5–30 vol%)^60,63,89^
Remanent magnetization (*M*_r_)	0–2 emu g^−1^ (1–15 wt%)^63,89^	1–121 emu g^−1^ (Nd_2_Fe_14_B, 7.5–30 vol%)^63,89^
Coercivity (*H*_c_)	0–100 Oe ^63,89^	500–5000 Oe ^63,89^
Magnetic field response time	<1 s (thin samples, 10–100 mT, low remanence)^90,91^	Dependence on magnetic orientation^90,91^
Applied magnetic field	10–100 mT ^63,91^	50–300 mT ^63,91^
Magnetic field guidance distance	0.5–2 cm (benchtop tests)^63,89^	>2 cm (stronger magnets.)^63,89^
Content of magnetic particles	0.25–15 wt% or 0.25–1% w/v^89^	0.005–0.05 mg mL^−1^ (MeCFO/GelMA-type); 7.5–30 vol% (Nd_2_Fe_14_B)^89^
Tensile strength	0.1–1.5 MPa (matrix- and test-mode-dependent)^90,91^	0.5–3 MPa ^90,91^
Deformation retention time	≈0 ^63,89^	1 h–3 d (programmed remanence; matrix-dependent)^63,89^
Biocompatibility	>85–95% (24–72 h)^88,91^	>80–90% (low-loading)^88,91^
MRI transverse relaxivity (*r*_2_)	100–300 mM^−1^ s^−1^ (Fe_3_O_4_-rich imaging systems)^60^	—

#### 2.2.1 Magnetic particles

Magnetic nanoparticles (MNPs) are the key functional components that impart magnetic responsiveness to MRHs. They are generally nanoscale magnetic materials (<100 nm), primarily composed of iron, iron oxides, nickel, cobalt, or their oxides.^92–94^ Below a critical size, MNPs exhibit size- and surface-dependent magnetic behavior distinct from bulk materials. Their high specific surface area, electrical and thermal conductivity, and ability to undergo magnetic enrichment, separation, and directional transport further broaden their functionality. Under high-frequency magnetic fields, they can also generate heat, supporting applications in drug delivery, tissue engineering, magnetic resonance imaging, and thermal therapy.

Common magnetic materials include Fe_3_O_4_, γ-Fe_2_O_3_, CoFe_2_O_4_, NiFe_2_O_4_, MnFe_2_O_4_, ZnFe_2_O_4_, Co, Fe, and Nd_2_Fe_14_B, which can be incorporated into hydrogel matrices to fabricate MRHs.^95–99^ Among them, Fe_3_O_4_ nanoparticles are particularly attractive because of their good biocompatibility, high magnetization, and ease of synthesis and functionalization, leading to widespread biomedical and pharmaceutical use.^99,100^

MNPs are commonly spherical and typically nanoscale, although magnetic composites may be larger. For example, MCMIPs exhibit a specific surface area of 192.19 m^2^ g^−1^, a pore volume of 0.80 cm^3^ g^−1^, and an average pore diameter of 1.99 nm, providing high and selective adsorption of cyanidin-3-*O*-glucoside (C3G).^101^ Ce-Fe_3_O_4_ MPs have an average particle size of 386 nm and a saturation magnetization of 43.13 emu g^−1^, while retaining their maximum adsorption capacity after five reuse cycles.^102^ MNP-NH_2_ particles are spherical, uniformly distributed, and approximately 20 nm in diameter; at a solid–liquid ratio of 1 : 40 (g), the corresponding system achieves the highest luteolin extraction efficiency.^103^

Based on magnetic behavior, magnetic materials can be broadly classified as diamagnetic, paramagnetic, ferromagnetic, ferrimagnetic, or superparamagnetic. Diamagnetic materials have a small negative magnetic susceptibility and develop an induced magnetic moment opposite to the applied field, whereas paramagnetic materials have a small positive susceptibility and weakly align with the field. In both cases, the induced magnetization disappears when the field is removed. Superparamagnetism occurs in sufficiently small single-domain ferro- or ferrimagnetic particles above their blocking temperature, where thermal fluctuations randomize the particle magnetic moments in the absence of a field. These particles exhibit high magnetic susceptibility under an applied field but negligible remanence after field removal.^22,24,25^ In contrast, ferromagnetic and ferrimagnetic materials may retain magnetization after removal of the external field and generally exhibit high magnetic susceptibility and saturation magnetization. Reducing ferro- or ferrimagnetic particles below a critical single-domain size can produce superparamagnetic behavior, which is advantageous for reversible and precise magnetic manipulation.

Magnetic composites formed by combining MNPs with other materials are generally larger than individual nanoparticles and provide high surface areas, good dispersion, and abundant adsorption sites. Typical architectures include core–shell, inverse core–shell, sandwich, and dispersed structures.^104^ In core–shell structures, the magnetic component forms the core and is coated by another material; in inverse core–shell structures, magnetic material forms the outer layer. Sandwich structures contain a magnetic intermediate layer between other materials, whereas dispersed structures distribute MNPs throughout the matrix.

#### 2.2.2 Soft magnetic hydrogels

Soft magnetic hydrogels are characterized primarily by low coercivity and low remanent magnetization rather than by a specific chemical composition. Superparamagnetic Fe_3_O_4_ nanoparticles are the most widely used magnetic phase, although sufficiently small ferrite nanoparticles, including CoFe_2_O_4_, can also exhibit superparamagnetic behavior. Under an applied magnetic field, these particles can generate magnetic forces or torques that induce hydrogel translation, orientation, or deformation. After field removal, their net magnetization largely decreases; however, recovery of the macroscopic hydrogel shape is additionally governed by the elasticity, viscoelasticity, and structural design of the polymer network. The low-remanence behavior of soft MRHs is advantageous for reversible actuation, injectable systems, magnetic guidance, and biomedical applications where persistent magnetic aggregation is undesirable. Their performance nevertheless depends strongly on particle size, concentration, dispersion, particle–polymer interactions, hydrogel stiffness, and the strength and gradient of the applied magnetic field. Therefore, magnetic responsiveness should be discussed together with the magnetic-field configuration and material-processing conditions rather than being represented by a universal response distance or field-strength range.

This reversible magnetic response enables soft MRHs to be remotely guided to specific locations. They also exhibit favorable biological properties and biocompatibility under alternating magnetic fields,^88^ supporting applications in targeted drug delivery, MRI contrast enhancement, tissue regeneration, magnetic hyperthermia, and multifunctional diagnostic and therapeutic platforms.^105^ Their fabrication methods are relatively well established. Common approaches include immersing hydrogels in ferrous chloride tetrahydrate and ferric chloride hexahydrate to generate magnetic particles within the network or directly mixing magnetic nanoparticles with hydrogel precursor solutions.^106^ Li *et al.*^90^ prepared Fe_3_O_4_/PVA MRHs through repeated freeze–thaw cycles. The resulting hydrogels showed improved tensile strength, elastic modulus, and overall mechanical performance, together with superparamagnetic behavior and rapid recovery after removal of the magnetic field. Similarly, superparamagnetic MRHs have been fabricated using ultrasmall cobalt ferrite nanoparticles.^60^

However, low remanence limits sustained and complex deformation because soft MRHs cannot maintain magnetization after the external field is removed. Their magnetic behavior depends strongly on particle type, size, and concentration, as on the field strength, field gradient, sample geometry, and source-to-sample distance.^62^ Consequently, response distance should be reported only together with the corresponding magnetic-field configuration and experimental geometry.

#### 2.2.3 Hard magnetic hydrogels

Hard magnetic hydrogels are prepared by incorporating hard magnetic particles, such as Co, Nd_2_Fe_14_B, and CoFe_2_O_4_, into hydrogel matrices. Unlike soft MRHs, they can retain magnetization after removal of the external magnetic field, enabling applications in targeted drug delivery, tissue engineering, magnetic sensing, and micro robotics. Li *et al.*^89^ prepared a CoFe_2_O_4_-based MRH using diethanolamine (DEA)-modified particles. The hydrogel retained magnetism after field removal, adsorbed heavy metal ions, and could be reused, demonstrating potential for treating heavy metal-contaminated wastewater. Similarly, Shi *et al.*^91^ fabricated a high-performance MRH using methacrylate-functionalized CoFe_2_O_4_. The resulting hydrogel exhibited high porosity, swelling capacity, shear-thinning behavior, and biocompatibility, indicating potential for postoperative repair following osteosarcoma resection. The retained magnetization of hard MRHs also allows prolonged magnetic localization, at the same time, it can provide greater flexibility in terms of magnetization programming and continuous driving. However, their practical use requires careful consideration of particle dispersion, hysteretic losses, mechanical fatigue, material-specific ion release, and biocompatibility. These issues become particularly important in long-term biomedical applications.

### 2.3 Preparation methods of magnetic-responsive hydrogels

MRHs are formed by incorporating magnetic particles into hydrogel polymer networks.^107–111^ They therefore combine the softness and high water content of hydrogels with the magnetic properties of embedded particles. Magnetic incorporation can also increase specific surface area and provide magnetic responsiveness, separation capability, and non-contact actuation. These features support applications in drug delivery, cell culture, magnetic hyperthermia, wastewater treatment, robotics, and related fields.

MRHs mainly consist of a hydrogel network and magnetic nanoparticles introduced through embedding or crosslinking. As summarized in Table 3, the principal fabrication methods are blending, *in situ* precipitation, and covalent bonding.^9,13,15,112^

**Table 3:** Preparation methods of hydrogels and magnetic-responsive hydrogels

Preparation method	Blending method	*In situ* precipitation method	Covalent bonding method
Source of magnetic particles	Fe_3_O_4_/CoFe_2_O_4_ nanoparticles	Nucleation within the Fe^2+^/Fe^3+^ network	Surface-functionalized Fe_3_O_4_/CoFe_2_O_4_ nanoparticles
Grain size	10–100 nm ^90^	5–30 nm (commonly about 10 nm)^60,62^	10–100 nm ^88,91^
Content	0.25–15 wt% ^90^	5–15 wt% ^60,62^	0.005–0.05 mg mL^−1^ ^88,91^
*M*_s_	3–20 emu g^−1^ ^90^	5–25 emu g^−1^ ^60,62^	3–20 emu g^−1^ ^88,91^
*M*_r_	0–2 emu g^−1^ ^90^	0–3 emu g^−1^ ^60,62^	0–15 (adjustable)^88,91^
*H*_c_	0–80 Oe ^90^	0–100 Oe ^60,62^	50–3000 Oe^88,91^
Elastic modulus	10–300 kpa	20–500 kpa ^60,62^	50–800 kpa ^88,91^
Particle loss rate	5–20%^90^	<5% ^60,62^	<1% ^88,91^

#### 2.3.1 Overview of hydrogel preparation methods

As shown in Fig. 5, hydrogel synthesis methods are broadly classified as physical or chemical crosslinking, with the appropriate strategy selected according to specific requirements and applications.^113–115^

**Fig. 5:**
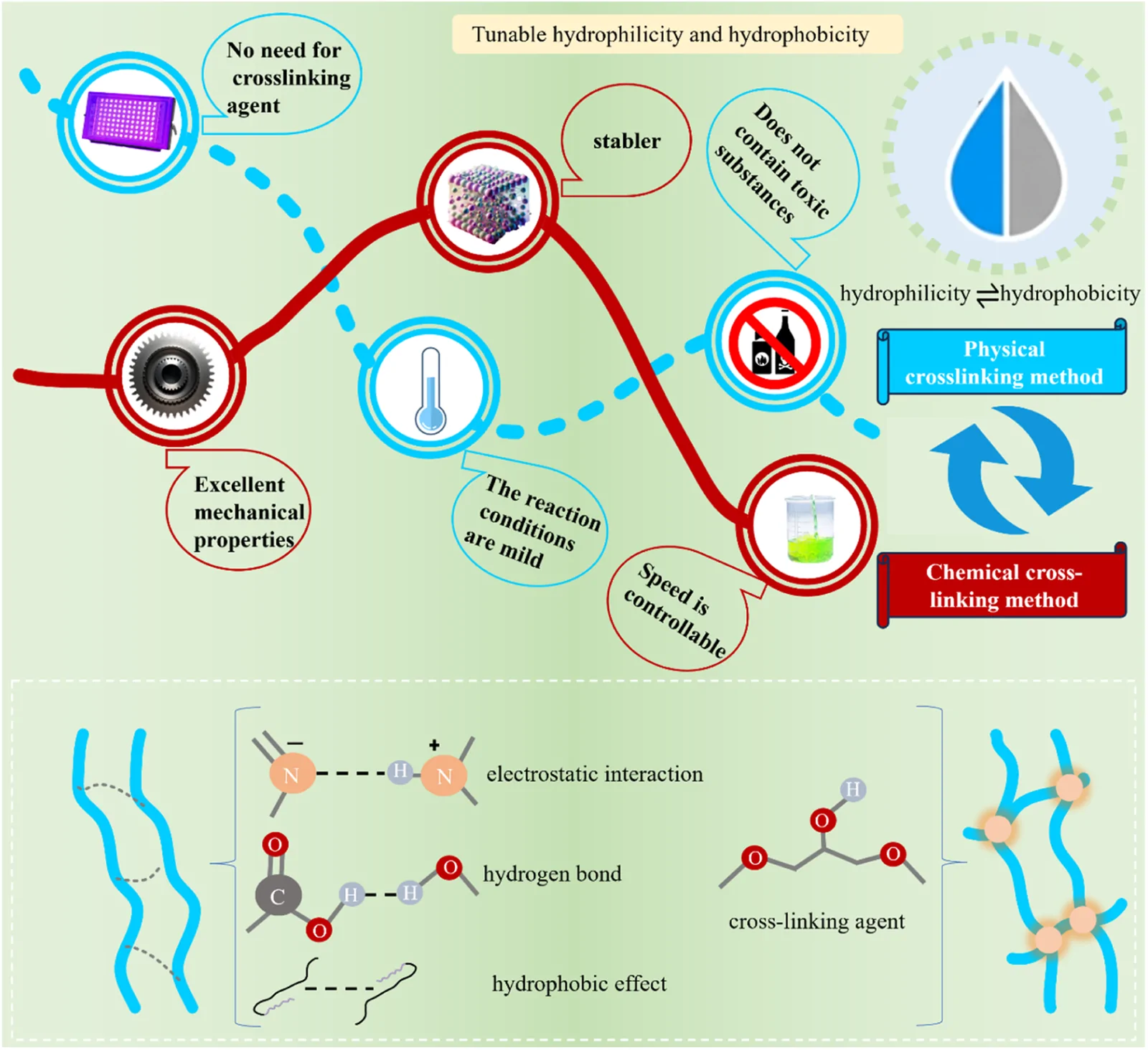
Hydrogel preparation methods.

Chemical crosslinking forms hydrogels through reactions between polymer chains, often with crosslinking agents. Common approaches include thermal, catalytic, photo-, and radiation-induced polymerization, enzymatic crosslinking, free-radical and condensation polymerization, Michael addition, Schiff base reactions, ionic crosslinking, and click chemistry.^77^ Chemically crosslinked hydrogels generally exhibit high mechanical strength and stability.^116^

Physical crosslinking forms hydrogels without conventional chemical crosslinkers through non-covalent interactions such as hydrogen bonding, van der Waals forces, crystallization, and hydrophobic interactions. Common methods include freeze–thaw cycling, hydrogen bonding, hydrophobic association, π–π stacking, physical entanglement, temperature-responsive crosslinking, solvent exchange, self-assembly, and associative crosslinking.^22,77^ Its mild conditions and limited use of potentially toxic reagents make it attractive for biomedical and environmental applications, while hydrogel properties can be tuned by regulating intermolecular interactions.^22,116^

#### 2.3.2 Blending method

The blending method involves directly dispersing pre-synthesized magnetic nanoparticles, typically prepared by co-precipitation or thermal decomposition, into a hydrogel precursor before gelation. Its simplicity and controllable particle characteristics make it one of the most widely used approaches for MRHs fabrication.^13^ Huang *et al.*^109^ prepared PVA/Fe_3_O_4_ MRHs by blending, obtaining increased saturation magnetization and potential for optically sensitive fiber measurements. Shi *et al.*^88^ similarly combined bisphosphonate-modified hyaluronic acid (HA-BP) with iron oxide nanoparticles to produce MRHs for tissue regeneration and anticancer applications.

A major limitation of blending is nanoparticle leakage and nonuniform distribution, particularly at low crosslinking densities. Surface modification and electrostatic stabilization have therefore been used to improve nanoparticle retention and dispersion.^74,117^ Narayana *et al.*^106^ prepared PAM-GA/magnetite nanocomposites in which the hydrogel network provided structural support and the nanoparticles increased surface area, enhancing swelling and adsorption of Cu(ii), Co(ii), and Ni(ii). Luís *et al.*^118^ incorporated liposomes and 12.7 nm magnetic nanoparticles into gelatin and crosslinked the system with glutaraldehyde, enabling magnetically triggered, non-thermal drug release. More broadly, blended MRHs can exhibit favorable superparamagnetism, magnetic responsiveness, thermal stability, and mechanical properties suitable for supporting cell growth.^119^ Increased surface porosity can further promote cell adhesion and growth, supporting their use as cartilage tissue-engineering scaffolds.^63^

From a structure–property perspective, blending provides the greatest processing simplicity but also the strongest dependence on nanoparticle surface chemistry and mixing conditions. At low particle loading, sufficiently stabilized nanoparticles may remain uniformly dispersed and can reinforce the polymer network through particle–polymer interactions. As particle concentration increases, however, magnetic dipole interactions and the high surface energy of nanoparticles can promote aggregation, which may generate mechanical defects, heterogeneous magnetic responses, and accelerated particle leakage. Increasing hydrogel crosslinking density can improve particle retention but may simultaneously reduce swelling and actuation strain. Therefore, particle loading in blended MRHs should be optimized rather than simply maximized to increase magnetization.

#### 2.3.3 *In Situ* precipitation method

The *in situ* precipitation method uses a preformed, swollen hydrogel as a reaction matrix in which Fe^2+^ and Fe^3+^ ions hydrolyze and precipitate under alkaline conditions to form magnetic nanoparticles. The hydrogel matrix is commonly prepared by free-radical polymerization.^120,121^ Key factors governing MRHs formation include hydrogel network properties, iron-ion concentration, and alkaline conditions.^122^ Zhao *et al.*^123^ prepared a hemicellulose-based MRH with enhanced superparamagnetism, swelling, saturation magnetization, compressive strength, pore uniformity, and adsorption capacity. Rana *et al.*^124^ synthesized alginate/gelatin MRHs with high saturation magnetization and drug-loading capability for cancer chemotherapy. Similarly, PDA–GO–Fe_3_O_4_ hydrogels exhibited high water content, hydrophilicity, tensile strength, load-bearing capacity, shear resistance, and biocompatibility, indicating potential as biomimetic soft-tissue substitutes.^125^

*In situ* precipitation generally promotes uniform nanoparticle distribution, but its alkaline conditions may damage the hydrogel network, introduce cytotoxic residues, and reduce biocompatibility. A recent approach involved immersing printed PNIPAM gels in Fe^2+^/Fe^3+^ solution followed by NaOH treatment to generate MRHs.^121^

Anionic polymer hydrogels can also serve as matrices for *in situ* co-precipitation.^126^ Negatively charged groups such as SO_3_^−^ and COO^−^ promote Fe^2+^/Fe^3+^ adsorption and distribution within the network. Inspired by magneto tactic bacteria, Gao *et al.*^127,128^ synthesized anisotropic Fe_3_O_4_ nanocrystals within an anionic PAMPS network. The SO_3_^−^ groups facilitated iron-ion adsorption and uniform distribution, while nanoparticle size and morphology could be tuned by hydrogel crosslinking density. Fe_3_O_4_ nanoneedles, nanorods, and nano-octahedra were further obtained by varying NaOH concentration under mild aqueous conditions.^128^

Wang *et al.*^129^ developed an alternative co-precipitation method in which magnetic nanoparticles formed during chitosan gelation through chelation between amino groups and iron ions. Hydroxide ions induced both chitosan deprotonation and nanoparticle crystallization, while chelation improved nanoparticle dispersion and hydrogel mechanical properties. Magnetic nanoparticle formation is strongly influenced by iron-ion concentration, precipitant concentration, and hydrogel crosslinking density. T. Miyazaki *et al.*^130^ showed that increasing chitosan crosslinking density produced larger but fewer magnetic nanocrystals because reduced swelling and fewer available amino groups limited iron-ion uptake. Compared with direct blending, *in situ* precipitation offers the important advantage that the hydrogel network acts as a confined reaction environment, which can suppress large-scale nanoparticle aggregation and improve spatial distribution. Nevertheless, the resulting particle size, number density, and magnetic properties are coupled to ion uptake and diffusion within the network. Increasing crosslinking density decreases hydrogel mesh size and swelling, which can restrict Fe^2+^/Fe^3+^ diffusion and alter nanoparticle nucleation and growth. Consequently, higher crosslinking density does not necessarily produce a larger number of smaller particles. Reaction pH is another important constraint because strongly alkaline conditions can hydrolyze chemically sensitive polymer networks and may introduce concerns regarding residual reagents for biomedical applications. Thus, *in situ* precipitation provides favorable particle confinement but requires simultaneous optimization of network chemistry, ion diffusion, precipitation conditions, and post-treatment purification.

#### 2.3.4 Covalent bonding method

Blending and *in situ* precipitation are widely used to fabricate MRHs, but the absence of covalent interactions may compromise nanoparticle retention and long-term stability. Covalent bonding was therefore introduced to address these limitations.^131^ In this method, magnetic nanoparticles are surface-functionalized and subsequently crosslinked with hydrogel precursors, allowing the functional groups to act as nanoscale crosslinkers and form stable covalent bonds with the polymer network.^95,132^ Huang *et al.*^62^ prepared a Fe_3_O_4_-containing gelatin/β-cyclodextrin magnetic nanohydrogel with a rough, porous surface that favored cell adhesion and proliferation. The principal advantage of covalent incorporation is therefore not simply higher particle retention but stronger and more controllable transfer of stress between the magnetic phase and the polymer network,^133^ this can improve mechanical stability during repeated deformation and reduce nanoparticle migration during long-term use.^134^ However, stronger particle–polymer coupling may also restrict local chain mobility and swelling if the particles effectively act as multifunctional crosslinkers. In addition, surface functionalization introduces additional synthesis, purification, and quality-control steps, and unreacted coupling agents may be undesirable for biomedical applications. Covalent bonding is therefore particularly attractive for systems requiring long-term particle retention and cyclic stability, whereas its greater fabrication complexity may be disadvantageous for large-scale or low-cost applications.^135^

From a preparation–performance perspective, the three major fabrication strategies exhibit distinct trade-offs. Blending provides the simplest processing route and is readily compatible with different hydrogel matrices; however, particle retention relies mainly on physical confinement or non-covalent interactions, making nanoparticle aggregation and leakage strongly dependent on surface chemistry, particle loading, and hydrogel mesh size. *In situ* precipitation uses the hydrogel network as a confined nanoreactor and can therefore improve particle distribution and spatial confinement. Nevertheless, nanoparticle nucleation and growth are highly sensitive to iron-ion concentration, pH, and crosslinking density, while alkaline treatment may damage chemically sensitive polymer networks. Covalent bonding provides stronger particle–polymer coupling, which generally favors nanoparticle retention, load transfer, and long-term structural stability, but requires additional surface-functionalization and coupling steps, increasing fabrication complexity and potentially introducing reagent-specific cytotoxicity. Thus, no single preparation strategy is universally optimal, and method selection should instead be guided by application-specific requirements for particle retention, mechanical stability, scalability, reproducibility, and biological safety.

#### 2.3.5 Preparation–structure–property relationships

The performance of MRHs is determined by coupled relationships among magnetic-particle size, particle concentration, surface chemistry, and hydrogel-network structure. Particle size affects both magnetic behavior and colloidal stability. Decreasing particle size can promote superparamagnetic behavior and increase specific surface area, but the resulting high surface energy also increases the tendency toward aggregation unless suitable surface stabilization is introduced. Particle loading generally increases the magnetic moment of the composite; for example, the saturation magnetization of PAM–Fe_3_O_4_ hydrogels increased from 3.41 to 9.05 emu g^−1^ as the Fe_3_O_4_ content increased from 5 to 15 wt%. However, excessive loading may introduce particle-rich defects, reduce network homogeneity, and alter swelling and mechanical deformation.

Surface functionalization regulates the particle–polymer interface. Hydrophilic or charged coatings can suppress aggregation and improve aqueous dispersion, whereas reactive functional groups can generate stronger particle–polymer bonding and reduce leakage. These benefits may be accompanied by dilution of magnetic material by non-magnetic coatings or increased synthesis complexity. Crosslinking density provides another key control parameter. Increasing crosslinking density generally improves modulus, dimensional stability, and particle retention, but decreases mesh size, swelling, ion diffusion, and sometimes actuation amplitude. During *in situ* precipitation, these effects also influence nanoparticle nucleation and growth because the availability and transport of iron ions depend on hydrogel swelling and accessible coordination sites. Therefore, optimization of MRHs requires simultaneous control of magnetic loading and polymer-network architecture rather than optimization of either component independently.

## 3 Surface modification of magnetic nanoparticles and its effects on MRHs performance

The type, size, surface chemistry, and dispersion state of magnetic nanoparticles strongly influence the magnetic, mechanical, interfacial, and biological properties of MRHs. Bare nanoparticles often exhibit high surface energy and may aggregate within aqueous precursor solutions or hydrogel networks. As shown in Fig. 6, surface modification with inorganic, organic, organic–inorganic hybrid, or small-molecule compounds can mitigate aggregation while introducing additional functionalities, thereby supporting the development of multifunctional MRHs.

**Fig. 6:**
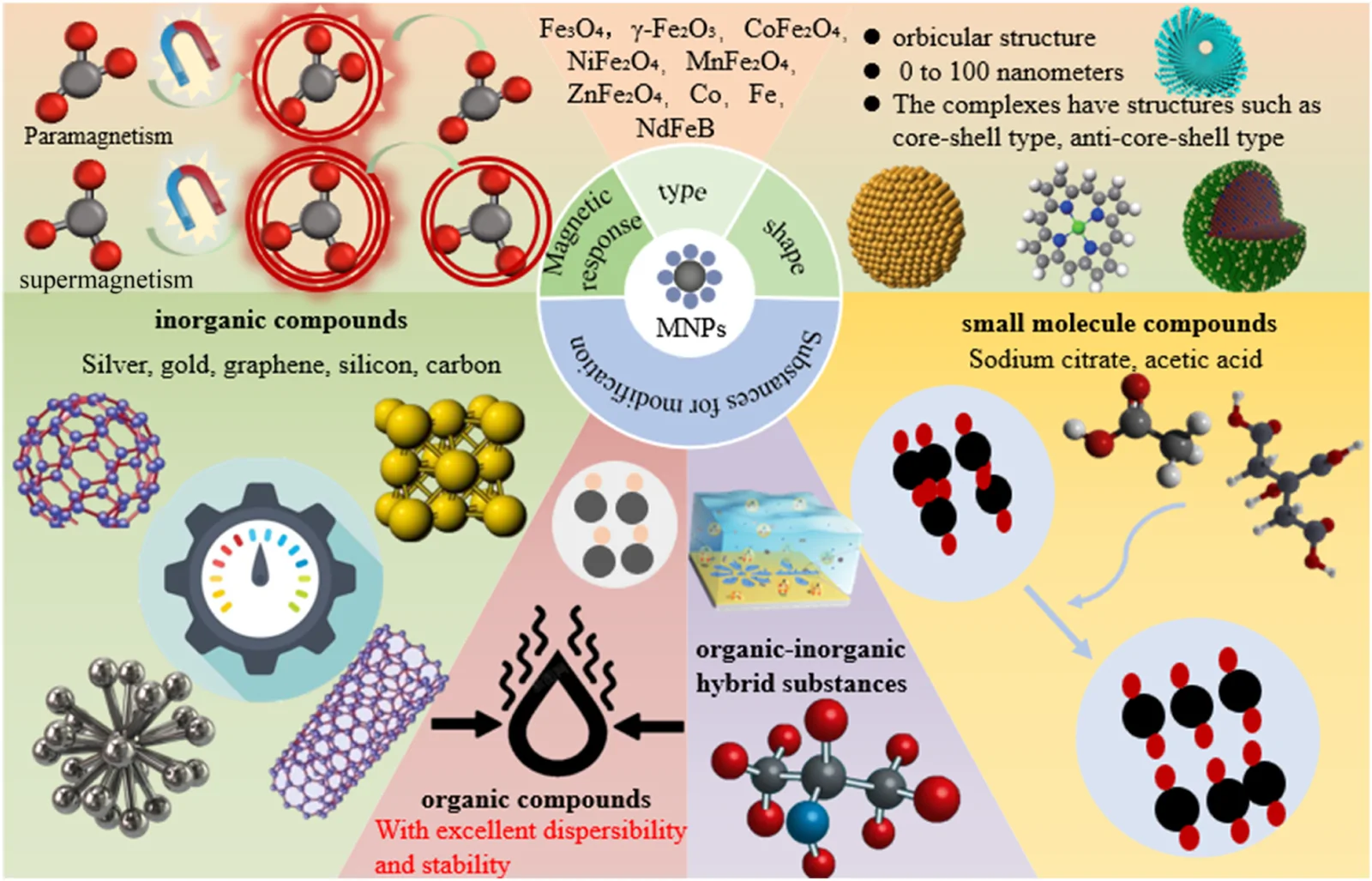
Overview of magnetic nanoparticles, their modification types, and effects.

Freshly synthesized magnetic nanoparticles exhibit superparamagnetism, high surface energy, a large surface-to-volume ratio, visible-light absorption, magnetic guidance, and good biocompatibility,^136^ the magnetic regime depends strongly on particle composition, size, crystallinity, and domain state. However, their high surface energy and reactivity can promote aggregation, whereas their hydrophilicity or hydrophobicity is primarily determined by surface chemistry.^26^ Fe_3_O_4_ nanoparticles are also susceptible to oxidation and acid/base corrosion, which can degrade their magnetic properties. Surface modification can improve their charge characteristics, wettability, stability, biocompatibility, and targeting ability, while introducing functional groups for ligand conjugation.^94^ As summarized in Table 4, Fe_3_O_4_ composite nanoparticles can be modified with organic, inorganic, or other materials. Their design should balance magnetic properties, particle size, biocompatibility, and biomacromolecule-binding capability.

**Table 4:** Effects of different magnetic nanoparticle modification strategies on the performance of magnetic-responsive hydrogels

Modifying type	Inorganic compound	Organic compounds	Organic–inorganic hybrid substances	Small molecule compound
Grain size	20–50 nm ^137^	15–40 nm ^129^	20–60 nm ^137,138^	10–30 nm ^138^
*M*_s_	20–50 emu g^−1^ ^137^	25–40 emu g^−1^ ^129^	25–55 emu g^−1^ ^137,138^	20–45 emu g^−1^ ^138^
Dispersed state	Even^137^	Highly uniform^129^	Extremely uniform^137,138^	Good^138^
Adsorption capacity	80–150 mg g^−1^ (adsorption)^137^	150–300 mg g^−1^ ^129^	300–700 mg g^−1^ ^137,138^	100–200 mg g^−1^ ^138^
Stability	>90% (5 times)^137^	>95% (5 times)^129^	90–95% (5 times)^137,138^	>85% (5 times)^138^

### 3.1 Inorganic compound modification and its effects

Common inorganic modifiers for Fe_3_O_4_ magnetic nanoparticles include silver, gold, graphene, silicon, and carbon. Inorganic modification typically involves coating Fe_3_O_4_ nanoparticles with inorganic materials or using them as seeds for surface growth, forming Fe_3_O_4_-based nanocomposites. As shown in Fig. 7a, these modifications improve chemical properties and introduce additional functionalities into the resulting MRHs.

**Fig. 7:**
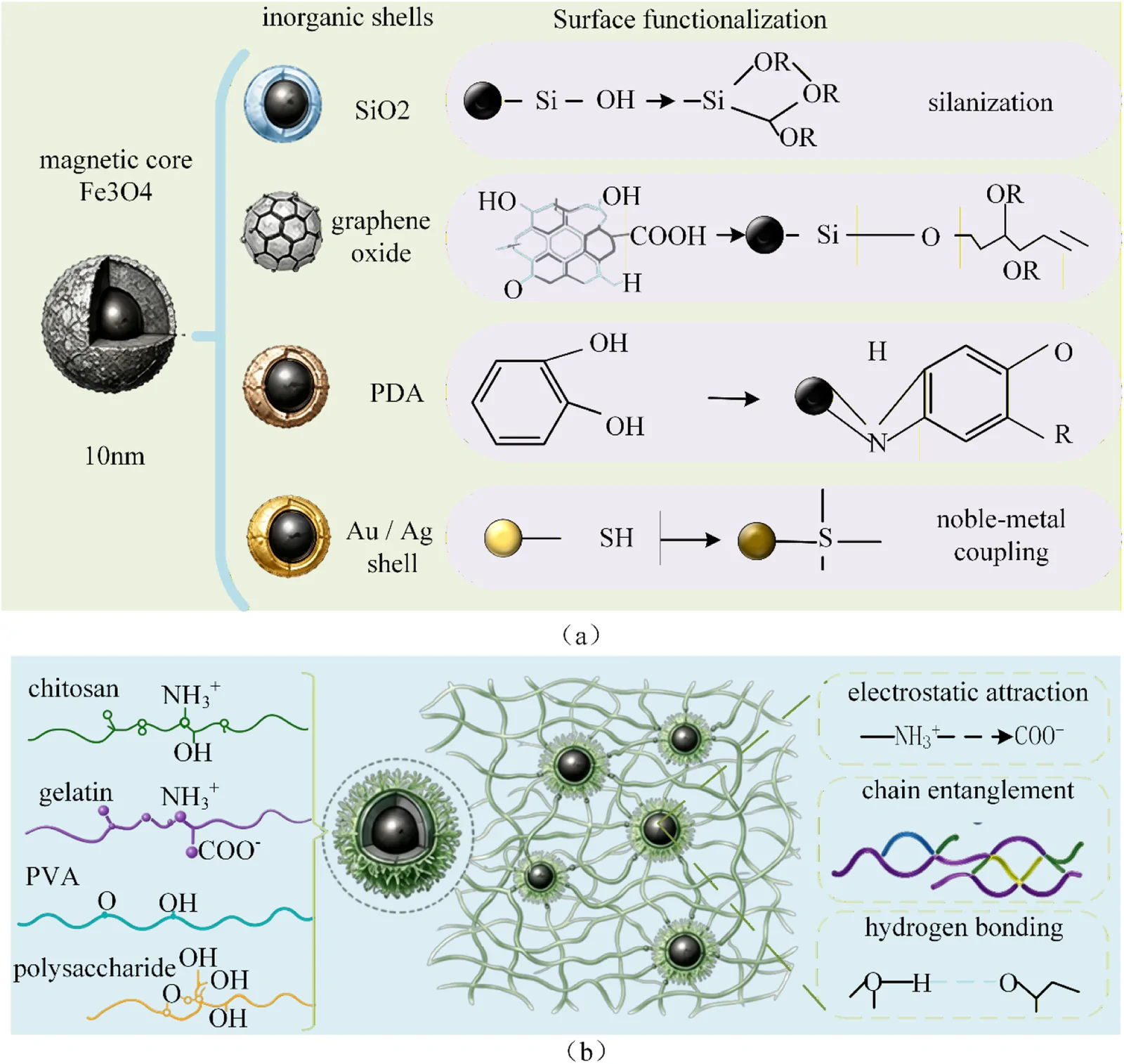
(a) Inorganic coating/interfacial coupling. (b) Organic polymer coating.

Zhang *et al.*^125^ prepared PDA–GO–Fe_3_O_4_ hydrogels by PDA-mediated *in situ* deposition of superparamagnetic Fe_3_O_4_ onto GO. The hydroxyl, carboxyl, carbonyl, and epoxy groups of GO, together with catechol groups on PDA, facilitated Fe_3_O_4_ formation under alkaline conditions while preserving the intrinsic properties of GO. The preparation process is shown in Fig. 8a1. Compared with the smooth layered morphology of pristine GO, PDA–Fe_3_O_4_–GO exhibited a rough surface densely covered with uniformly distributed nanoparticles (Fig. 8a2), improving hydrogel lubricity, load-bearing capacity, and compressive strength. In addition, Katarzyna *et al.*^139^ incorporated silver-modified magnetic nanoparticles into a hydrogel matrix. As shown in Fig. 8c3, the nanoparticles exhibited a strong UV-visible absorption peak at approximately 435 nm, indicating the formation of Fe@O@Ag. The modification reduced cytotoxicity while introducing antibacterial activity. Increasing the magnetic nanocomposite content also enhanced hydrogel adsorption, while nanoparticle incorporation markedly increased surface roughness and promoted adsorption of incubation solutions. The resulting magnetic hydrogel was successfully explored as a drug-delivery carrier.

**Fig. 8:**
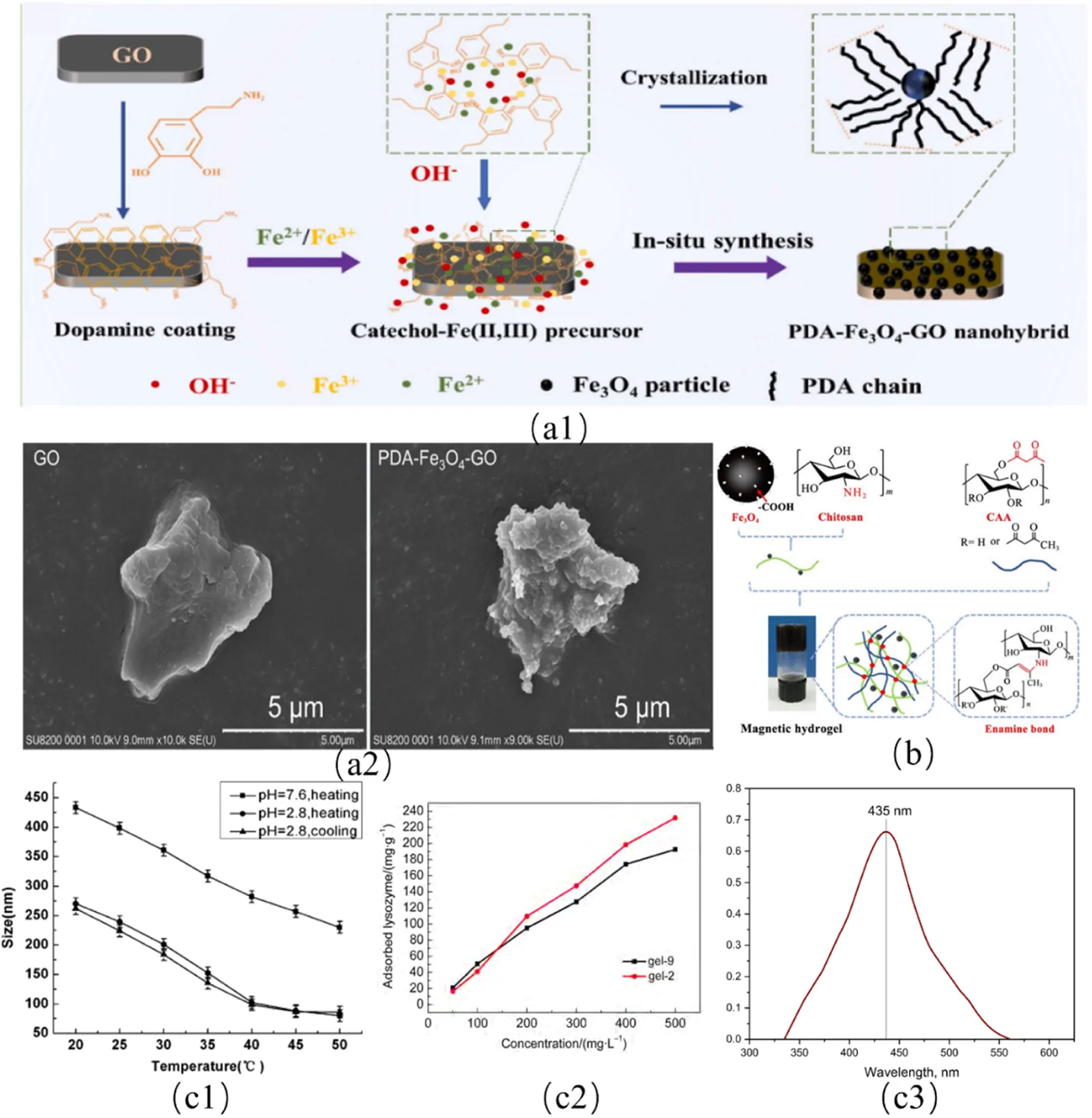
(a1) Preparation process of magnetic-responsive hydrogels^125^ Copyright 2026 Elsevier. (a2) Schematic of the surface structures of GO and PDA–Fe_3_O_4_–GO^125^ Copyright 2026 Elsevier. (b) Schematic of magnetic-responsive hydrogel preparation^140^ Copyright 2020 Wiley Online Library. (c1) Effect of temperature on the size of magnetic-responsive hydrogel nanospheres^141^ Copyright 2011 Elsevier. (c2) Effect of initial lysozyme concentration on the adsorption capacity of magnetic-responsive hydrogels^137^ Copyright 2014 Elsevier. (c3) Analysis of the UV-visible spectrum of silver-modified magnetic nanoparticles^139^ Copyright 2023 MDPI.

Inorganic modification of magnetic nanoparticles can enhance MRHs performance through synergistic effects between components. These modifiers introduce additional functionalities while preserving magnetic responsiveness and remote actuation, thereby expanding the potential of MRHs in precision medicine, environmental remediation, and related applications.

### 3.2 Organic compound modification and its effects

Inorganic modification alone is often insufficient to prevent nanoparticle aggregation and improve dispersion; therefore, organic modifiers are widely used to functionalize Fe_3_O_4_ nanoparticles. Common modifiers include polyethyleneimine (PEI), 3-aminopropyltriethoxysilane (APTES), amines, diethanolamine, carboxymethyl cellulose/polyacrylic acid/starch, 3-(trimethoxysilyl)propyl methacrylate (MPS),^142^ oleic acid, agarose, hexamethylene diisocyanate (HMDI),^143^ DETA-lignin and cellulose,^144^ amino groups,^145,146^ carboxyl groups, polyethylene glycol (PEG), polyvinyl alcohol (PVA), and chitosan, among others, as shown in Fig. 7b.

Organic modification can enhance the multifunctionality of MRHs, including their magnetic responsiveness, electroactivity, conductivity, and mechanical properties.^119^ Gao *et al.*^145^ introduced amino groups, including –NH_2_, –NH(CH_3_), and –N(CH_3_), onto Fe_3_O_4_ nanoparticles to develop a magnetic-responsive semi-convertible hydrogel (MSCH). Under a magnetic field, electrostatically bound magnetic particles drive reversible gel-to-gel–sol transitions, while the covalent network maintains structural integrity. This behavior enables the hydrogel to pass through confined channels or obstacles, supporting applications in soft robotics, actuation, and targeted drug delivery. Liu *et al.*^140^ uniformly dispersed carboxyl-modified Fe_3_O_4_ nanoparticles in a chitosan matrix to prepare magnetic polysaccharide hydrogels (Fig. 8b). The resulting hydrogel exhibited pH responsiveness, self-healing, stability for 72 h under physiological conditions (37 °C), injectability, and magnetic-field-induced deformation, indicating potential for biomedical applications.

Organic modification can improve the dispersion and stability of magnetic nanoparticles in MRHs. Common modifiers include 3-aminopropyltriethoxysilane (APTES),^147^ agarose,^148^ carboxymethyl cellulose/polyacrylic acid/starch,^149^ and high-magnetization polyethylene glycol (PEG),^150^ These modifications can suppress nanoparticle aggregation while preserving sufficient magnetism for external-field-assisted separation. Notably, Fe_3_O_4_@agarose nanoparticles impart both magnetic and pH responsiveness, enabling sustained release triggered by either stimulus.

Tao *et al.*^134,151,152^ prepared polyethyleneimine-modified magnetic hydrogel nanocomposites (PEI-mHNCs) by radical copolymerization of polyacrylamide–acrylic acid with PEI-modified magnetic nanoparticles. Steric hindrance from PEI chains reduced nanoparticle aggregation, while the porous three-dimensional network promoted crystal-violet diffusion and adsorption. Abdeen *et al.*^71^ functionalized carbonyl iron (CI) particles with amino propyl triethoxy silane (APTES) or 3-(trimethoxysilyl)propyl methacrylate (TMSPM). APTES improved long-term particle stability, whereas TMSPM enabled covalent attachment to the hydrogel. Amine-functionalized hydrogels showed improved stability compared with untreated gels, which dissolved within 10 days. However, TMSPM modification induced particle aggregation, likely because of heterogeneous copolymerization, and repeated magnetic actuation could cause particle leaching.

Organic modification can also enable structural control of MRHs. Zhong *et al.*^142^ modified Fe_3_O_4_ nanoparticles with 3-(trimethoxysilyl)propyl methacrylate (MPS), improving their dispersion and stability. Magnetic-responsive three-dimensional hydrogel microstructures of approximately 10 µm were then fabricated by two-photon polymerization (TPP), demonstrating the potential to construct functional biomedical microdevices with tunable geometries.

Overall, combining organically modified magnetic nanoparticles with functional polymers improves nanoparticle dispersion, stability, and structural tunability, supporting applications in targeted drug delivery, soft robotics, biosensing, and water remediation.

### 3.3 Organic–inorganic hybrid modification and its effects

Silane coupling agents, such as 3-aminopropyltriethoxysilane, are widely used as organic–inorganic modifiers for Fe_3_O_4_ nanoparticles. These modifications improve nanoparticle dispersion and hydrogel compatibility while introducing active surface groups, thereby enhancing biocompatibility, redox activity, adsorption, and drug-delivery performance, as shown in Fig. 9a. Li *et al.*^137^ modified Fe_3_O_4_ with vinyl triethoxysilane in a water–ethanol solution. Hydrolyzed silanol groups interacted with surface hydroxyl groups on Fe_3_O_4_ and subsequently formed covalent bonds through condensation, introducing vinyl groups for the preparation of a hemicellulose-based MRH. As shown in Fig. 8c2, the magnetic hydrogel gel-2 exhibited higher adsorption capacity than the non-magnetic gel-9, together with pH sensitivity, magnetic responsiveness, superparamagnetism, and enhanced oxidation properties. Alireza *et al.*^138^ functionalized Fe_3_O_4_ with amine-terminated silane coupling agents and combined this modification with CaCl_2_-mediated dual crosslinking to produce a redox-responsive MRH for drug delivery.

**Fig. 9:**
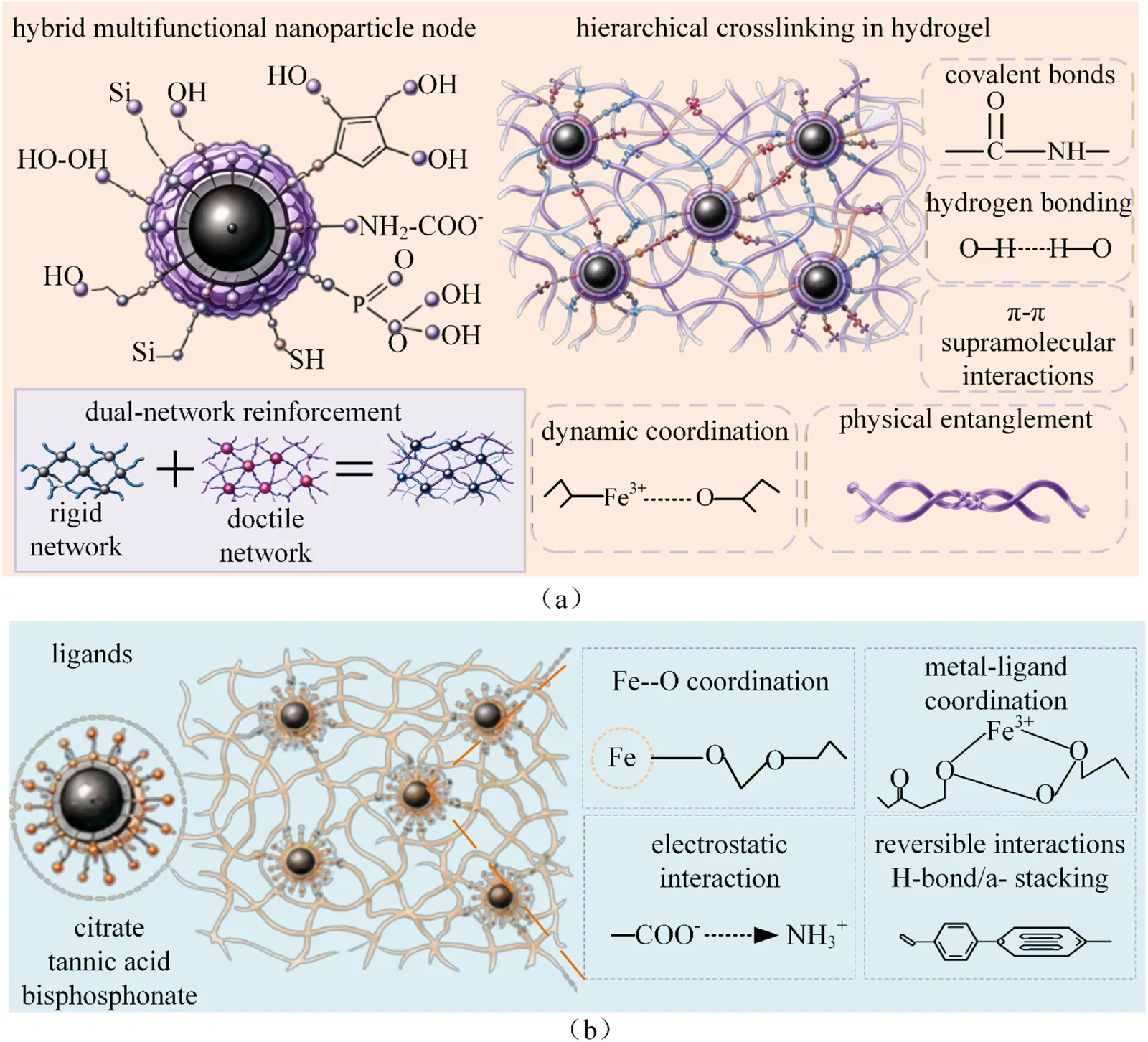
(a) Organic–inorganic hybrid crosslinking. (b) Small-molecule ligand anchoring.

Organic–inorganic hybrid modification forms stable, functionalized interfacial layers on magnetic nanoparticles through strong covalent interactions. This approach enhances MRHs stability while introducing active functional sites, thereby broadening their application potential.

### 3.4 Small molecule modification and its effects

Small molecules are also used to modify Fe_3_O_4_ nanoparticles, with common examples including sodium citrate^153^ and acetic acid. These modifiers can improve nanoparticle dispersion and hydrophilicity, as shown in Fig. 9b. Hao *et al.*^154^ modified Fe_3_O_4_ nanoparticles with trisodium citrate, which grafted onto the particle surface and improved their hydrophilicity, aqueous dispersion, and magnetic responsiveness. The modified nanoparticles were incorporated into 3D-printed magnetic soft grippers for soft-robotic applications. Fan *et al.*^141^ physically encapsulated sodium citrate-modified Fe_3_O_4_ nanoparticles within hydrogel nanospheres. As shown in Fig. 8c1, the resulting MRHs nanospheres effectively loaded doxorubicin (DOX) and enabled rapid release at pH 5.3 and 37 °C. The DOX-loaded nanospheres also showed enhanced antitumor activity, supporting their potential for anticancer drug delivery.

Small-molecule modification offers good dispersibility, simple processing, and retention of magnetic responsiveness. However, its limited functionality and relatively weak interfacial bonding restrict applications requiring high stability, mechanical performance, or biocompatibility. In such cases, secondary modification or alternative surface-functionalization strategies may be required.

Overall, surface modification expands MRHs from simple magnetic systems to multifunctional materials. Inorganic modifiers mainly introduce additional functions, organic modifiers improve compatibility and biological integration, organic–inorganic hybrids enhance interfacial bonding and stability, and small molecules provide simple and rapid modification. The optimal strategy, or combination of strategies, should therefore be selected according to the requirements of the intended application.

## 4 Properties of magnetic-responsive hydrogels

MRHs combine the high-water content, softness, and biocompatibility of conventional hydrogels with rapid magnetic-field responsiveness. Their overall performance is governed by multiple factors.

### 4.1 Magnetic responsiveness

The most distinctive property of MRHs is their responsiveness to external magnetic fields. Magnetic energy can be converted into physical or chemical changes, including deformation, directional alignment, and thermal effects, mainly through field-responsive behavior and magnetic hysteresis.

Magnetic-field responsiveness enables MRHs to undergo rapid deformation, such as bending, contraction, and elongation, as well as directional movement. When embedded magnetic particles are magnetized, magnetic-field gradients generate translational forces, whereas uniform fields exert magnetic torques that align the particles and induce macroscopic deformation. This enables contactless and remotely controlled actuation for applications such as soft robotics, drug delivery, and artificial muscles. Xuan *et al.*^155^ developed a 3D-printable MRH with tunable stiffness, adhesion, and magnetic responsiveness, as shown in Fig. 10a. Tests conducted in air, water, and under magnetic actuation demonstrated flexible field-driven movement (Fig. 10b) and magnetothermal behavior (Fig. 10c). Room-temperature hysteresis measurements of PAM and PAM–Fe_3_O_4_ hydrogels showed lower magnetization than pure Fe_3_O_4_ but narrow hysteresis loops, indicating low coercivity. As shown in Fig. 10d, saturation magnetization increased with Fe_3_O_4_ content, demonstrating that increasing magnetic particle loading effectively enhances MRHs magnetization.

**Fig. 10:**
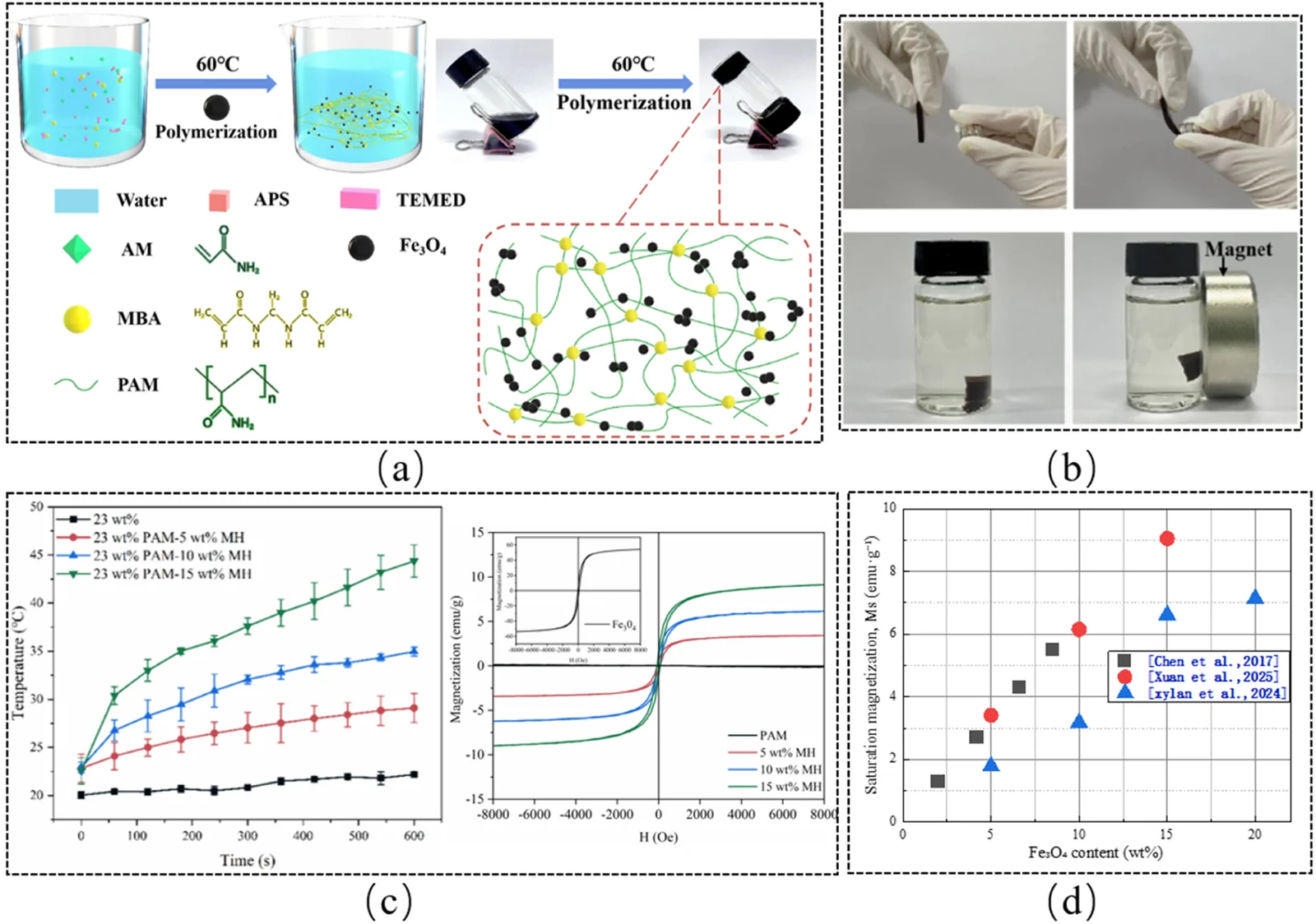
(a) Preparation process of magnetic-responsive hydrogels^155^ Copyright 2025 MDPI. (b) Behavior of hydrogels in air, water, and under magnetic field induction^155^ Copyright 2025 MDPI. (c) Magnetothermal response of magnetic-responsive hydrogels and their hysteresis loops at room-temperature^155^ Copyright 2025 MDPI. (d) The influence of magnetic particle content on the saturation magnetization (*M*_s_).

Hysteresis is an intrinsic property of magnetic materials in which magnetization depends on both the current magnetic field and its previous history. Incorporating magnetic particles therefore introduces hysteretic behavior into MRHs. For non-superparamagnetic particles, the hysteresis loop has a finite area, reflecting energy dissipation as heat during magnetization–demagnetization cycles. Partrick *et al.*^156^ reported that stronger interactions among magnetic nanoparticles increased hysteresis by restricting particle rotation.

The magnetic behavior of MRHs is governed by both field responsiveness and hysteresis. Field responsiveness determines deformation and actuation under an applied magnetic field, whereas hysteresis reflects magnetic retention and energy dissipation. Both properties can be regulated by controlling the type, size, concentration, and distribution of magnetic particles, enabling MRHs to be tailored for specific applications.

### 4.2 Mechanical properties

Mechanical properties describe a material's resistance to deformation and failure under external loading and are commonly characterized by strength, stiffness, toughness, ductility, and fatigue resistance. In MRHs, these properties are governed by the magnetic nanomaterials, polymer-chain characteristics, particle–polymer interactions, and crosslinking density.^109^

Magnetic nanoparticles can act as physical crosslinking sites or form strong interfacial interactions with polymer chains, thereby improving hydrogel strength, modulus, toughness, and fatigue resistance. Under a magnetic field, magnetic forces and torques can induce nanoparticle movement or alignment, which is transferred through the polymer network to produce reversible deformation. Mechanical performance is therefore critical for applications such as drug delivery and wound dressings.

Inspired by the ordered structure of natural cartilage, Zhang *et al.*^125^ developed a PDA–GO–Fe_3_O_4_ hydrogel using magnetic-field-induced network alignment and nanoparticle reinforcement. Under an external magnetic field, PDA–GO–Fe_3_O_4_ nanocomplexes aligned within the hydrogel matrix (Fig. 11d), producing a fracture strength of 11.0 MPa, elongation at break of 453%, tensile modulus of 16.0 MPa, and fracture toughness of 37.43 MJ m^−3^. These values increased by 57%, 40%, 57%, and 146%, respectively, compared with the non-oriented hydrogel. The hydrogel also exhibited ∼33.8% stress relaxation at 70% strain and a peak compressive strength of 20 MPa. These improvements were attributed to the ordered network and reversible hydrogen bonding, which promoted particle dispersion, load transfer, and energy dissipation. As shown in Fig. 11a, tensile strength increased with magnetic-particle content. MRHs can also retain self-healing capability after mechanical loading.^62^

**Fig. 11:**
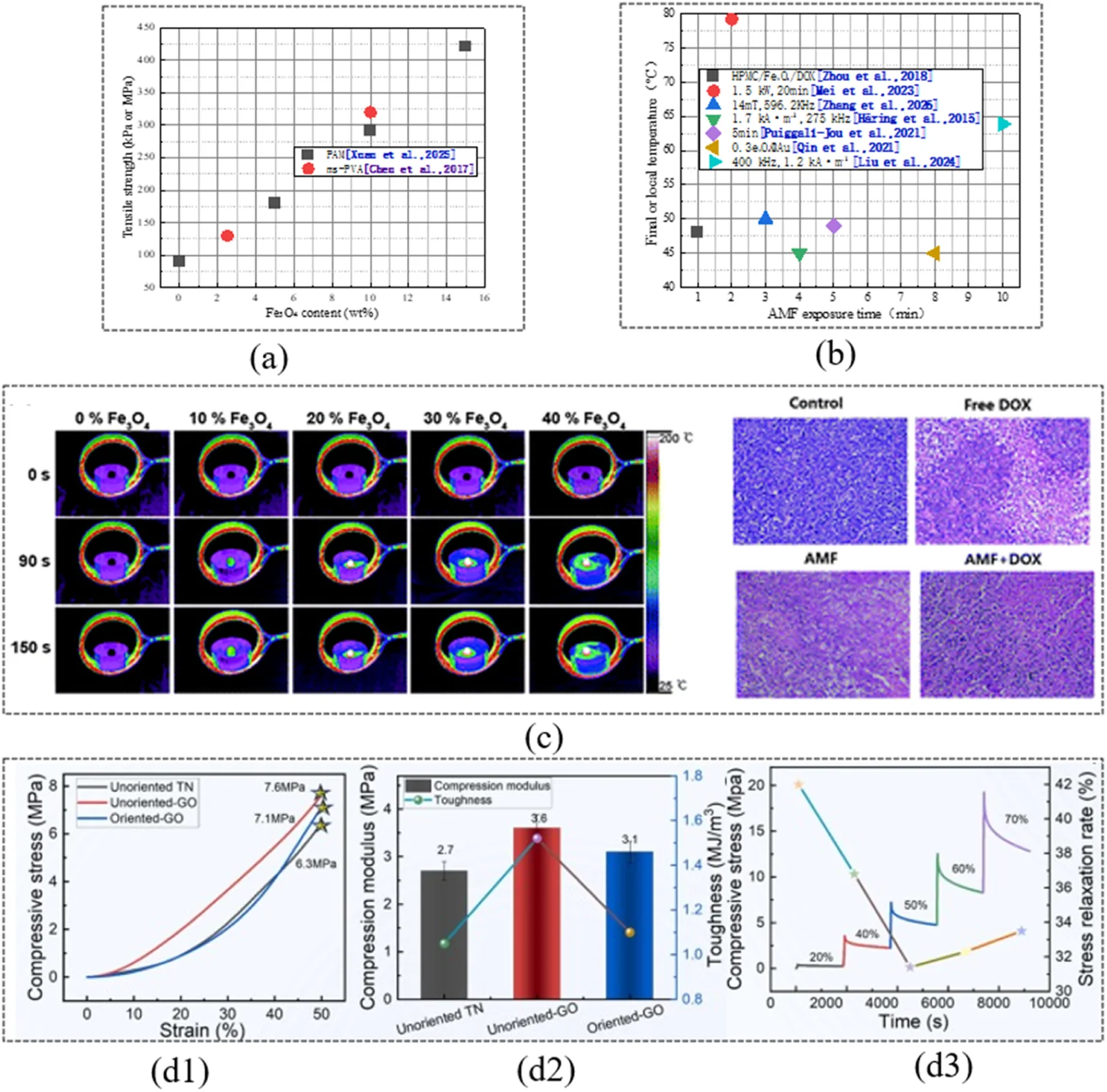
(a) The influence of magnetic particle content on tensile strength. (b) The final temperature of the magnetic-responsive hydrogel under the action of alternating magnetic field. (c) Thermal images of magnetic-responsive hydrogels with different Fe_3_O_4_ particle contents at various time points and staining comparison of tumors in mice three days post-treatment^58^ Copyright 2017 The Royal Society of Chemistry. (d) Stress testing of magnetic-responsive hydrogels^125^ Copyright 2026 Elsevier.

Current strategies for improving hydrogel mechanical properties include constructing double-network structures,^157,158^ introducing strong hydrogen bonds or topological networks,^159^ fiber reinforcement, optimizing gelation conditions and formulation ratios, ionic coordination, salting-out, and lateral cutting. Incorporating magnetic nanoparticles can also reinforce hydrogel networks^158^ and these strategies are similarly applicable to MRHs. Magnetic nanoparticles provide additional interaction sites within the polymer network. Under mechanical loading, weak interfacial interactions can reversibly dissociate or rearrange to dissipate energy while preserving network integrity.^160–162^ Nanoparticle incorporation can also alter thermal stability, swelling, water retention, saturation magnetization, and pore structure. Reversible particle–polymer interactions may further impart self-healing, shear-thinning, thermoelastic, and enhanced viscoelastic properties.^9^

Xuan *et al.*^155^ prepared a 3D-printable MRH with tunable stiffness, adhesion, and magnetic responsiveness using a one-pot method. Hydrogen bonding between nanoclay and PAM reinforced the network, while Fe_3_O_4_ nanoparticles introduced additional interaction sites and enhanced network connectivity. By varying acrylamide (AM) and Fe_3_O_4_ concentrations, the elastic modulus was tuned from 43 to 277 kPa, tensile strength from 93 to 421 kPa, and toughness from 243 to 1400 kJ m^−3^.

### 4.3 Thermal responsiveness

The thermal responsiveness of MRHs, or magnetothermal effect, arises when embedded magnetic nanoparticles generate heat under an alternating magnetic field (AMF). This heating can trigger conformational changes in thermosensitive polymer chains, leading to reversible changes in hydrogel volume or permeability and enabling temperature-controlled or pulsatile drug release.

Magnetothermal responsiveness is mainly exploited in biomedical applications such as tumor therapy and tissue engineering. As shown in Fig. 11b and c, AMFs can deliver energy to magnetically responsive materials without requiring direct optical access. However, the resulting heating efficiency depends on magnetic-particle composition, concentration, hysteresis and relaxation behavior, field amplitude and frequency, exposure duration, heat dissipation, sample geometry, and surrounding tissue properties. MRHs can localize magnetic nanoparticles and thereby combine magnetothermal heating with on-demand drug release to enhance anticancer efficacy,^58,163^ but their thermal output must be carefully controlled to avoid unintended heating of adjacent tissues. Free magnetic nanoparticles used for hyperthermia may suffer from rapid clearance, short retention, limited efficacy, repeated dosing requirements, and potential overheating of surrounding tissues. As shown in Fig. 13b, many MRHs reach approximately 45–80 °C within 1–10 min, although their temperature profiles are not necessarily monotonic. Incorporating magnetic nanoparticles into hydrogel matrices can prolong local retention and improve therapeutic performance.

The three-dimensional, water-rich hydrogel network can localize magnetic nanoparticles, reduce unintended thermal exposure, and provide scaffolds that support cell adhesion, migration, and differentiation,^67,164^ MRHs can also combine magnetically guided drug delivery with magnetothermal therapy to improve treatment efficacy.^165^ Moreover, controlled alignment of magnetic nanoparticles can enhance magnetothermal performance.^166^

### 4.4 Biocompatibility and translational safety

Biocompatibility is a fundamental requirement for biomedical materials and reflects their ability to perform safely and appropriately within a biological environment. Its evaluation mainly considers biosafety and bio functionality, requiring low toxicity together with the ability to support the intended physiological function. Natural polymers such as chitosan, fibrin, collagen, and hyaluronic acid are widely used for MRHs fabrication because of their low toxicity, biocompatibility, and biodegradability,^167^ Other biodegradable polymers, such as polylactic acid, can also degrade into metabolites that are processed by the body.

Many MRHs based on these materials exhibit low cytotoxicity. As shown in Fig. 12a, cell viability remained above 50% over the investigated concentration range up to 3 mg mL^−1^, indicating that the IC_50_ exceeded the highest tested concentration under these specific experimental conditions. This result suggests limited acute cytotoxicity within the tested concentration range but should not be interpreted as evidence of universal biocompatibility,^60^ In addition, Huang *et al.*^62^ also developed an MRH with a rough, microporous surface favorable for cell adhesion and growth. Co-culture with bone marrow mesenchymal stem cells (BMSCs) showed high cell viability, while pulsed electromagnetic field (PEMF) stimulation further promoted cell proliferation, as shown in Fig. 12b. These favorable biological properties support the use of MRHs in tissue engineering, tumor therapy, cancer-related applications, and controlled drug delivery.

**Fig. 12:**
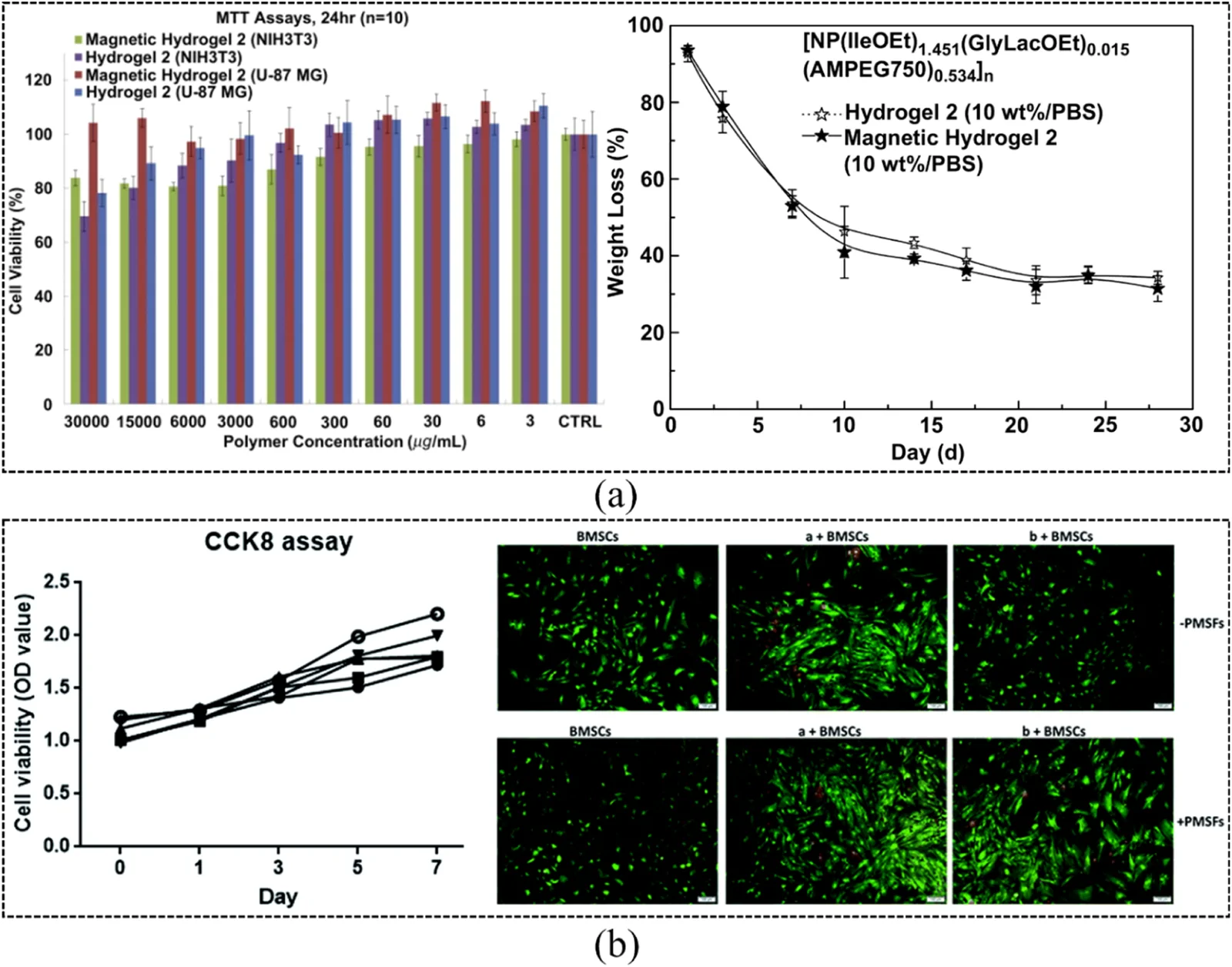
(a) Cytotoxicity assessment of magnetic-responsive hydrogels^167^ Copyright 2019 Elsevier. (b) Live/dead cell staining^62^ Copyright 2020 Royal Soc Chemistry.

**Fig. 13:**
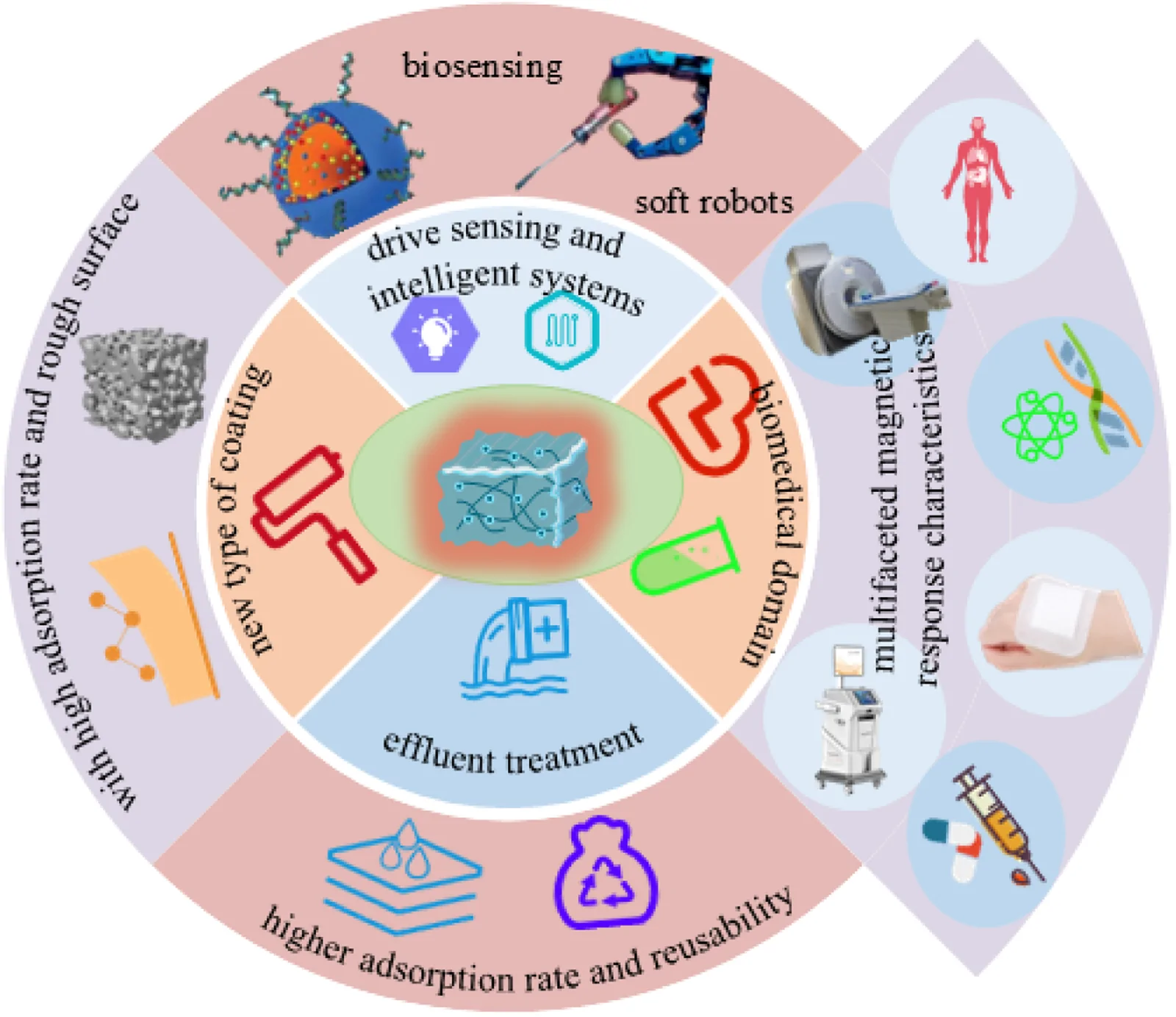
Applications of magnetic-responsive hydrogels.

Beyond short-term cytocompatibility, the translational safety of MRHs depends on the long-term fate of both the hydrogel matrix and magnetic components. Degradation of the polymer network may expose or release magnetic nanoparticles, while corrosion or dissolution of magnetic phases may result in metal-ion release. Therefore, particle leakage, degradation kinetics, inflammatory responses, hemocompatibility, and local tissue reactions should be systematically evaluated under application-relevant conditions. These issues are particularly important for long-term implants and for magnetic materials containing elements other than iron.

Biodistribution and clearance are also important considerations. Following degradation or nanoparticle release, magnetic components may be taken up by macrophages and accumulate in clearance-related organs. Consequently, long-term *in vivo* studies should evaluate particle retention, metal-ion release, organ accumulation, clearance pathways, and chronic inflammatory responses rather than relying exclusively on short-term cell-viability measurements. Magnetic-field exposure itself should also be considered as a dose-dependent factor. For alternating magnetic field applications, field amplitude, frequency, exposure duration, temperature evolution, and repeated-exposure cycles should be reported. Excessive magnetothermal heating or repeated mechanical stimulation may cause unintended tissue damage. In addition, sterilization procedures may alter hydrogel crosslinking, swelling, mechanical properties, or nanoparticle surface chemistry, and should therefore be incorporated into preclinical evaluation.

### 4.5 Designability and processability

MRHs are magnetically responsive materials with high designability and processability, supporting applications in biomedicine and soft robotics. Their composition, microstructure, magnetic behavior, and shape can be tailored for specific requirements. For example, selecting magnetic fillers such as Fe_3_O_4_ or Fe_2_O_3_ and controlling their size, morphology, distribution, and surface functionality can regulate magnetic responsiveness,^112^ Fabrication methods such as blending and *in situ* precipitation can further influence nanoparticle dispersion and reduce aggregation or leakage.^109^ Together with favorable mechanical adaptability and biocompatibility, these tunable features broaden the application range of MRHs.^33^

## 5 Applications of magnetic-responsive hydrogels for composite smart material

As a multifunctional smart material, MRHS, as shown in Fig. 13, have broad applications in biomedical fields, wastewater treatment, actuation and sensing in intelligent systems, and other emerging areas.

### 5.1 Biomedical field

As a material responsive to external stimuli, MRHS exhibit multiple magnetic-responsive properties in biomedical applications, they can move under an applied magnetic field, and the heat generated by MRHs can also be utilized in hyperthermia, thermally controlled drug delivery, and soft actuators.^52^

#### 5.1.1 Drug delivery

Drug efficacy depends not only on the drug itself but also on the delivery route and release profile. Owing to their biocompatibility, porous structure, and tunable drug encapsulation and release behavior, hydrogels are widely used as drug-delivery carriers.^56,168–171^ However, conventional hydrogels mainly rely on passive diffusion and matrix degradation, which may limit release control and therapeutic efficiency. Controlled delivery is therefore required to maintain effective drug concentrations while reducing side effects.^169^ Compared with conventional passive hydrogel carriers, MRHs provide an additional means of externally regulating localization and/or drug release. A magnetic-field gradient can exert forces on magnetically responsive carriers and thereby promote their accumulation or retention near a target region, whereas alternating magnetic fields can generate localized heating that triggers temperature-sensitive drug release. These approaches may increase local drug exposure and reduce off-target exposure under appropriately optimized conditions. However, magnetic targeting is not independent of tissue depth. Targeting efficiency decreases when the magnetic-field gradient at the target becomes insufficient and is governed by the magnetic-field strength and gradient, source-to-target distance, particle magnetization and concentration, carrier size, blood flow, and tissue geometry. Therefore, successful magnetic targeting requires coordinated optimization of both the MRH formulation and the external magnetic-field configuration.^118,172–177^

Studies have demonstrated improved targeting efficiency using MRHs,^178^ Mushtaq *et al.*^105^ co-encapsulated flurbiprofen and superparamagnetic iron oxide nanoparticles in a dual-balloon hydrogel system for rheumatoid arthritis treatment (Fig. 14a and d). Magnetic targeting improved drug encapsulation and delivery reduced arthritic symptoms in rats by 27.83%, and increased motor activity by 85%. External magnetic fields can also provide remote, contactless control of drug release,^179^ for example, Wang *et al.*^180^ fabricated concentric-layer chitosan-based MRHs by alternating alkali treatment, enabling low-frequency alternating-magnetic-field-triggered drug release, as shown in Fig. 14c.

**Fig. 14:**
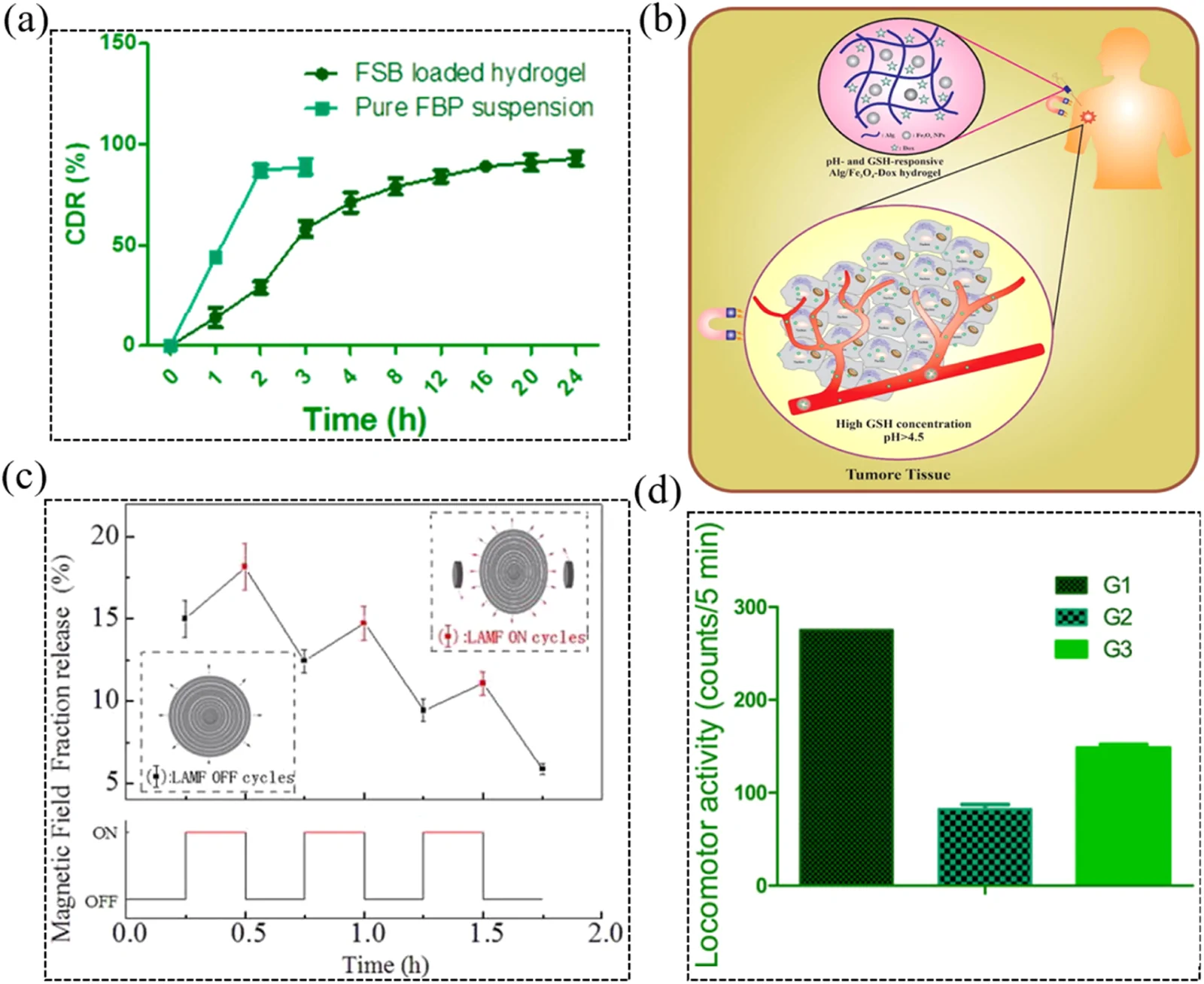
(a) *In vitro* drug release curve of drug-loaded magnetic-responsive hydrogels^105^ Copyright 2024 Elsevier. (b) Principle of drug delivery^138^ Copyright 2023 Elsevier. (c) The effect of magnetic field stimulation on drug release curve^180^ Copyright 2013 Elsevier. (d) The effect of drug-loaded magnetic-responsive hydrogels on joint diameter in *in vivo* experiments^105^ Copyright 2024 Elsevier.

MRHs have increasingly been combined with pH-responsive strategies to improve controlled and targeted drug delivery. Hu *et al.*^148^ fabricated an ale can-*g*-poly(VA-*co*-HEA)/Fe_3_O_4_@agarose MRH in which mildly acidic conditions or an external magnetic field accelerated doxorubicin hydrochloride (DOX) release. The released DOX retained high cytotoxicity toward A549 cells, supporting its use in magnetically targeted delivery. Reza *et al.*^149^ developed pH-sensitive CMC/PAA/St-Fe_3_O_4_ nanocomposite hydrogel beads for oral delivery of 5-FU and DOX. Under simulated gastrointestinal conditions, the beads suppressed drug release in the acidic stomach while promoting delivery to the colon. Combining pH sensitivity with magnetic guidance enables site-specific release, reduces exposure to healthy tissues, and increases local drug concentrations at target sites.

Magnetic nanoparticles can also promote sustained and controlled drug release.^167^ Tharushi *et al.*^181^ developed a carboxymethyl cellulose/Fe_3_O_4_ hydrogel crosslinked with fumaric acid for sustained curcumin delivery. Alireza *et al.*^138^ prepared an MRH for sustained and targeted DOX delivery (Fig. 14b). Although free DOX showed greater early cytotoxicity at 24 and 48 h, the hydrogel drug-delivery system exhibited higher anticancer activity after 72 h because of controlled release. Similarly, Mohammad *et al.*^167^ incorporated 5-fluorouracil (5-FU) into chitosan/polyacrylic acid/Fe_3_O_4_ hydrogels, achieving controlled release and improved dosage stability.

#### 5.1.2 Magnetic hyperthermia

Hyperthermia has emerged as an important adjunct to chemotherapy, radiotherapy, and surgery for cancer treatment. It kills tumor cells by raising local temperature. Tumor cells are particularly susceptible within 42–46 °C, where protein and DNA denaturation can induce apoptosis or increase sensitivity to radiation and anticancer drugs, whereas temperatures above 46 °C generally cause necrosis.^182,183^ Because excessive heating may also damage healthy tissue, 42–46 °C is widely regarded as an appropriate therapeutic range.^184,185^

Magnetic nanoparticles generate heat under high frequency alternating magnetic fields through Brownian and Néel relaxation. MRHs can therefore serve as localized magnetothermal platforms, and previous studies have also shown their potential as long-term magnetic resonance imaging systems for applications such as brain tumor diagnosis.^60^ Under AMF stimulation, MRHs can generate sufficient heat for effective tumor ablation.^186,187^ Injectable MRHs are especially attractive because they can be delivered minimally invasively into deep tumor tissues.^23^ Qian *et al.*^188^ developed an injectable silk fibroin/iron oxide nanocube MRH that was delivered to deep tissue by ultrasound-guided intervention (Fig. 15a). Under an AMF of 30 kA m^−1^ and 312 kHz for 15 min, the hydrogel showed effective remote heating and antitumor activity, while the silk fibroin matrix restricted nanoparticle diffusion and reduced thermal injury to surrounding healthy tissue. Thermosensitive injectable MRHs have also been designed to undergo sol–gel transition after injection, thereby limiting nanoparticle leakage. As shown in Fig. 15c, these systems can also enhance drug release under magnetic stimulation, with stronger release under higher magnetic fields.^86,189^

**Fig. 15:**
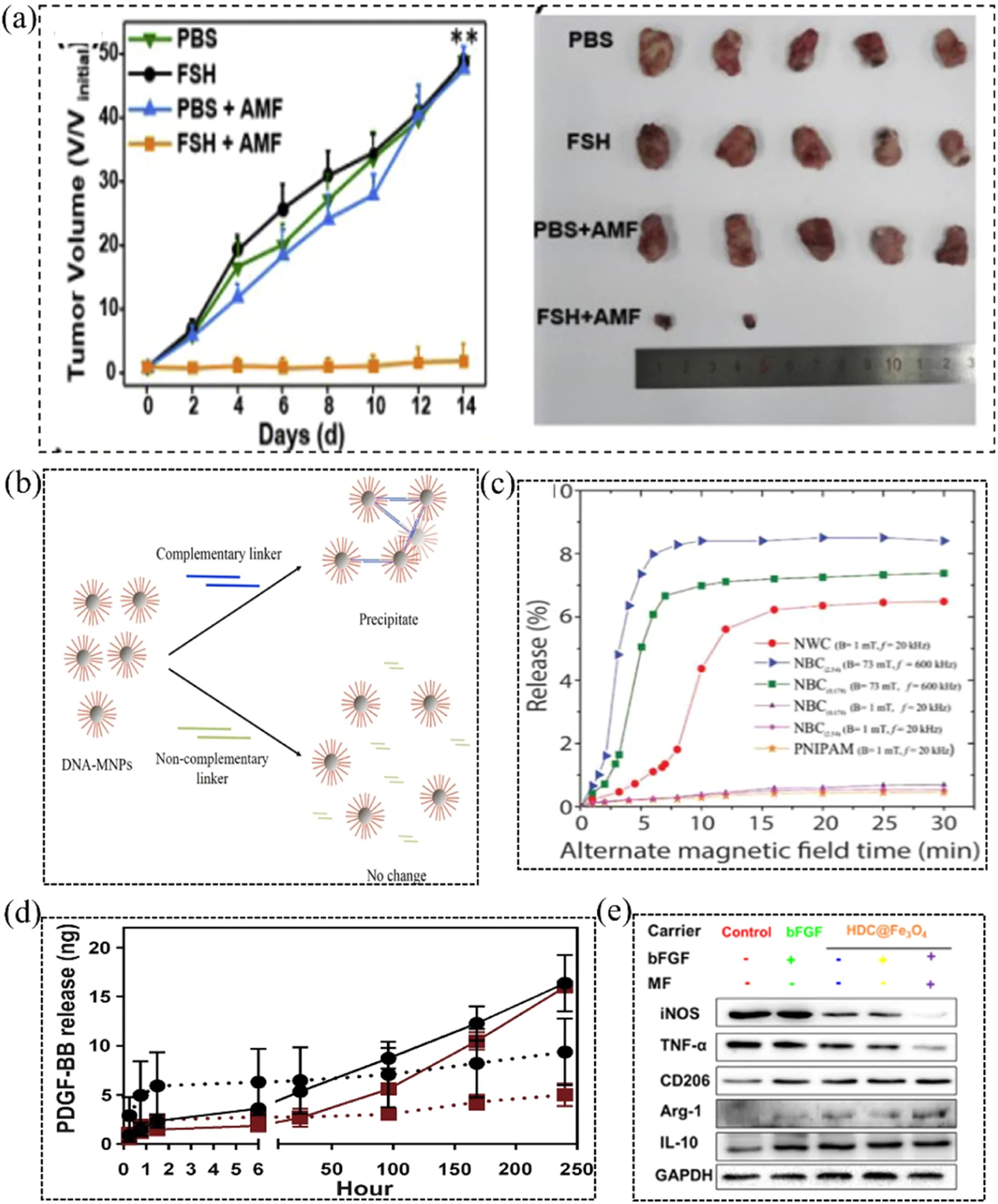
(a) The tumor growth curve of mice injected with magnetic-responsive hydrogel and the tumor staining images before and after treatment^188^ Copyright 2020 Elsevier. (b) Schematic diagram of magnetic-responsive hydrogel after adding complementary and non-complementary DNA strands^190^ Copyright 2017 Amer Chemical Soc. (c) Drug release behavior of magnetic-responsive hydrogels under low- and high-power magnetic field stimulation^188^ Copyright 2020 Elsevier. (d) The drug release curve of MA-CS in magnetic-responsive hydrogels over 250 hours (ref. 191) Copyright 2018 Elsevier. (e) Inflammatory modulation by bFGF-HDC@ Fe_3_O_4_ magnetic-responsive hydrogel^192^ Copyright 2021 Amer Chemical Soc.

Achieving effective tumor therapy at the clinically relevant hyperthermia temperature of 42 °C while overcoming heat-stress resistance remains challenging. Wu *et al.*^193^ developed a magnetic hydrogel nanozyme (MHZ) composed of PEGylated nanoparticles complexed with α-cyclodextrin. In the acidic tumor microenvironment, the nanozyme exhibited peroxidase-like activity that generated reactive oxygen species (ROS), thereby increasing oxidative stress and enhancing magnetic hyperthermia. Its shear-thinning and magnetothermal phase-transition properties enabled injection and effective distribution within tumor tissue. Fe_3_O_4_ nanoparticles provided localized heating at 42 °C while simultaneously catalyzing ROS generation, producing a synergistic therapeutic effect that reduced tumor resistance to heat stress without causing significant damage to normal tissues.

#### 5.1.3 Biomolecular separation and DNA-based magnetic hydrogels

Magnetic hydrogels can serve as functional platforms for biomolecular separation because their porous polymer networks provide adsorption or capture sites, while the magnetic component facilitates rapid recovery from complex media. Early magnetic hydrogel microspheres were primarily investigated for protein adsorption and purification. Ding *et al.*^194–196^ prepared thermosensitive Fe_3_O_4_/P(St-NIPAM) hydrogel microspheres by dispersion polymerization and investigated their adsorption/desorption of human serum albumin (HSA). The microspheres enabled rapid magnetic separation without agglomeration and could be recycled, supporting applications in protein and enzyme purification, recovery, and immobilization.

Ma *et al.*^190^ incorporated DNA-modified magnetic nanoparticles into DNA hydrogels to construct a DNA–MNP MRH, as shown in Fig. 15b. The hydrogel responded to magnetic fields, temperature, enzymes, and AMF, enabling remote deformation, directional movement, and jumping. It could also adhere to and integrate with pure DNA hydrogels, facilitating their magnetic manipulation while maintaining reversible sol–gel behavior. These properties support potential applications in cell culture, tissue engineering, biomedicine, and DNA-related separation or extraction.

Alpsoy *et al.*^197^ developed hydrogel beads containing magnetic nanoparticles and DNA capture probes for selective nucleic-acid capture. Target DNA could be captured from complex biological matrices, followed by magnetic separation and washing for downstream molecular analysis. More recently, magnetic hydrogel beads containing nucleic-acid capture probes have been developed for DNA isolation from complex biological matrices. The hydrogel network provides probe-functionalized capture sites, whereas embedded magnetic nanoparticles facilitate magnetic separation and washing, thereby enabling enrichment and purification of target DNA for downstream molecular analysis.

#### 5.1.4 Tissue engineering

Owing to their good biocompatibility, MRHs are widely used in tissue engineering, including tissue repair and scaffold fabrication.^86,198^. Adequate compressive strength and mechanical integrity are essential for scaffolds to withstand repeated loading while preserving tissue function.^199^ However, achieving mechanical properties comparable to native tissues remains challenging. Under an external magnetic field, embedded magnetic nanoparticles can generate localized mechanical stimulation at the hydrogel–cell interface, providing dynamic three-dimensional cues to cells.^100,200–203^

Huang *et al.*^63^ prepared a PVA/nano-calcium phosphate/magnetic nanoparticle composite hydrogel scaffold for cartilage tissue engineering. Combining the scaffold with pulsed electromagnetic fields (PEMFs) promoted mesenchymal stem-cell chondrogenic differentiation^62^ and upregulated aggrecan, SOX9, and collagen II expression, improving cartilage repair in animal models.^204^ In the reported experimental system, PEMF exposure at 100 mT and 40–70 Hz for 20 min promoted chondrogenic differentiation.^204^ These parameters should be regarded as study-specific stimulation conditions rather than generally optimal magnetic-field parameters, and the underlying mechanobiological mechanisms remain to be clarified.

Elsa *et al.*^191^ developed a multifunctional methacrylated chondroitin sulfate hydrogel containing iron-based superparamagnetic nanoparticles and platelet lysate. Its swelling, degradation, and growth-factor release could be regulated by an external magnetic field, as shown in Fig. 15d. PDGF-BB exhibited an initial burst followed by sustained release for up to 10 days without hyaluronidase (Haase). Electromagnetic-field stimulation accelerated release, particularly in the presence of Haase, supporting its potential for interface tissue engineering.

#### 5.1.5 Magnetic resonance imaging (MRI)

Magnetic resonance imaging (MRI) is a non-invasive technique that provides high-contrast soft-tissue images by detecting signals from hydrogen nuclei in a magnetic field. Its safety, high tissue penetration, and diverse imaging sequences make it widely used for clinical diagnosis and monitoring. However, overlapping relaxation times among tissues can limit image contrast, necessitating contrast agents. Fe_3_O_4_-based superparamagnetic nanoparticles are attractive MRI contrast agents because of their magnetic properties and biocompatibility. Incorporating these particles into hydrogels can enhance MRHs imaging performance.^205,206^

MRI also enables longitudinal, non-invasive monitoring of biomaterial degradation and tissue regeneration *in vivo*. This approach reduces the need for repeated histological sampling, minimizes inter-individual variability, and facilitates adjustment of tissue-engineering protocols.^207^ MRHs can therefore provide sustained MRI contrast and support long-term clinical monitoring. However, concerns regarding uncontrolled heat generation *in vivo* may limit certain applications.^111^

#### 5.1.6 Wound dressing

Wound-healing materials should provide antibacterial and anti-inflammatory activity, biocompatibility, and support collagen deposition, angiogenesis, and tissue remodeling.^208,209^ Unlike traditional dry dressings, hydrogels maintain a moist environment favorable for tissue regeneration because of their high water-retention capacity.^210–213^

A tannic acid (TA)-bridged CFO/PVA MRH has been developed by incorporating cobalt ferrite (CFO) nanoparticles into a PVA matrix. TA and Co^2+^ provide biochemical cues, while a static magnetic field (SMF) generates mechanical stimulation. Under SMF exposure, the TA bridge transfers magnetically induced deformation from CFO nanoparticles to the PVA network, increasing surface morphological changes that promote cell adhesion and proliferation while providing antibacterial activity. *In vivo* studies further showed accelerated full-thickness wound healing, mainly through enhanced early vascularization induced by magnetically driven mechanical stimulation.^214^

Wu *et al.*^192^ developed a bFGF-loaded magnetic composite hydrogel (bFGF-HDC@Fe_3_O_4_). As shown in Fig. 15e, the hydrogel stabilized bFGF and enabled sustained release, promoting macrophage polarization toward the anti-inflammatory M2 phenotype. SMF stimulation further enhanced cell proliferation and accelerated wound healing.

Self-assembling peptide hydrogels represent a complementary class of bio functional hydrogel matrices for biomedical applications.^215^ Their nanofibrous structures can provide injectable, cell-interactive, and intrinsically antibacterial environments. For example, β-hairpin peptide hydrogels and lipopeptide-based hydrogels have demonstrated intrinsic antibacterial activity and favorable biocompatibility, while injectable self-assembling peptide nanofiber hydrogels have been investigated for chronic wound regeneration.^216–218^ Although these peptide systems are not inherently magnetic, integrating magnetic nanoparticles into peptide-based matrices may provide a promising strategy for combining biological functionality with remote magnetic actuation or magnetothermal regulation. Such hybrid systems would nevertheless require careful evaluation of nanoparticle effects on peptide self-assembly, mechanical stability, and biological activity.

### 5.2 Environmental engineering field

Environmental remediation represents an important non-biomedical application of MRHs because the hydrogel network can provide abundant adsorption sites and mass-transfer pathways, while the magnetic component enables convenient solid–liquid separation and adsorbent recovery. Their practical performance is governed by adsorption-site density, pore structure, swelling behavior, magnetic recovery efficiency, regeneration stability, and nanoparticle retention.

#### 5.2.1 Wastewater treatment

Wastewater may contain heavy metals, acids, alkalis, salts, sulfides, and pathogenic microorganisms, posing environmental and health risks if untreated.^219–222^ Compared with conventional hydrogels, MRHs can be magnetically recovered after adsorption and often exhibit high adsorption efficiency and reusability.^223–227^ They have therefore been widely investigated for wastewater treatment. MRHs microspheres can adsorb metal ions and proteins,^106^ while magnetic separation enables rapid removal of low concentrations of pathogenic microorganisms.^219,226,228,229^

Li *et al.*^137^ prepared a hemicellulose-based MRH using silane-modified Fe_3_O_4_ for protein adsorption. Adsorption behavior was described using Freundlich and Temkin isotherm models, indicating potential for protein-containing wastewater treatment. Tao *et al.*^134,151,152^ developed PEI-modified MRHs nanocomposites with rough, porous surfaces suitable for removing cationic dyes from water. In another study, an APTES-modified Fe_3_O_4_/chitosan-*g*-polyacrylic acid MRH reached adsorption equilibrium within 20 min and achieved maximum adsorption capacities of 308.84 mg g^−1^ for Cd^2+^ and 695.22 mg g^−1^ for Pb^2+^, as shown in Fig. 16c. The material retained high adsorption capacity after five adsorption–desorption cycles, while Fe_3_O_4_ enabled magnetic recovery.^147^

**Fig. 16:**
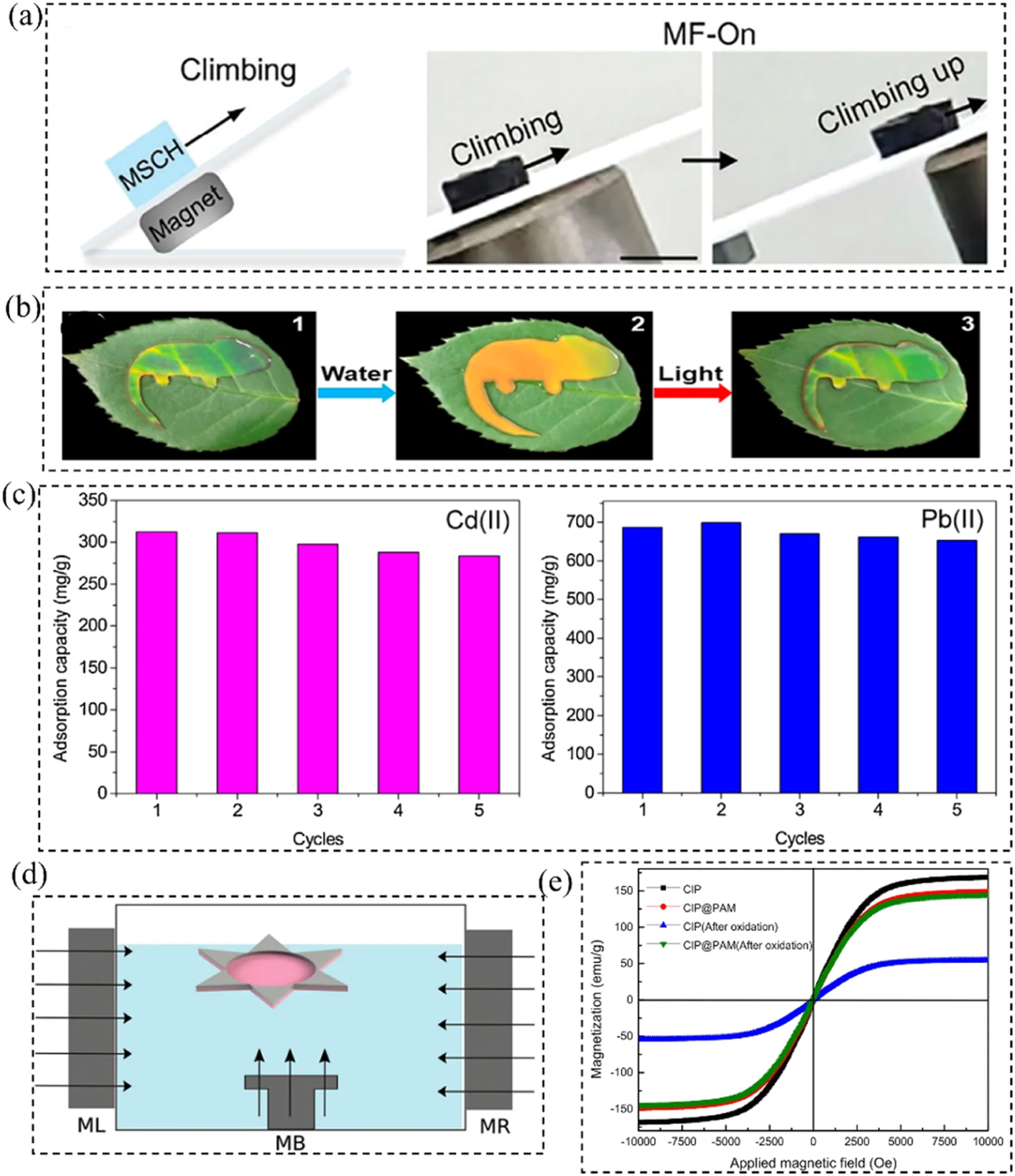
(a) Demonstration of semi-converted magnetic-responsive hydrogel climbing under the influence of a magnetic field^230,231^ Copyright 2019 Amer Chemical Soc, Copyright 2025 Royal Soc Chemistry. (b) Adaptive camouflage performance of chameleon-like magnetic-responsive hydrogel^232^ Copyright 2022 Amer Chemical Soc. (c) Adsorption amounts of Cd^2+^ and Pb^2+^ as a function of adsorption–desorption cycles^147^ Copyright 2016 Elsevier. (d) Schematic diagram of remote-controlled soft mechanical motion^233^ Copyright 2019 Wiley. (e) Hysteresis loop of the sample before and after oxidation in a water–air environment for 15 days (ref. 234) Copyright 2022 Elsevier.

### 5.3 Intelligent systems and sensor technology field

MRH-based actuation and sensing have become important research directions in intelligent material systems. Magnetic fields can remotely regulate the position, deformation, orientation, and internal microstructure of MRHs, enabling applications in soft robotics, adaptive actuators, and sensing platforms. Their performance depends on magnetic force or torque generation, hydrogel stiffness, structural anisotropy, response speed, and cyclic stability.^12^

#### 5.3.1 Soft robotics

Soft robots are flexible systems that can adapt to unstructured environments and interact safely with humans, supporting applications in artificial muscles, artificial skin, and minimally invasive surgery.^235^ MRHs are particularly attractive for untethered soft-robotic applications because magnetic actuation does not require a direct physical connection or optical line of sight. Nevertheless, the achievable force and controllability depend on magnetic-field strength and gradient, distance from the magnetic source, magnetic loading, and device geometry.^110,145,236^

Tognato *et al.*^233^ developed a biocompatible MRH in which low-intensity magnetic fields aligned iron oxide nanoparticles into tunable filamentous structures within a gelatin methacrylate matrix. Cells could align along with these filaments, and the system was compatible with bioprinting to fabricate three-dimensional structures (Fig. 16d), supporting applications in soft robotics and bio actuation. Gao *et al.*^145^ developed an MRH capable of magnetically induced gel–sol transitions. Magnetic forces disrupted gelatin triple helices and produced a surface sol layer, while the covalent network maintained structural integrity, enabling the hydrogel to pass through confined channels and obstacles (Fig. 16a).

Magnetothermal effects can also drive soft actuators. In bilayer systems composed of an elastomer and a magnetic hydrogel layer, high-frequency AMF heating above the lower critical solution temperature (LCST) induces hydrogel contraction and bending.^230^ This mechanism has been used to produce flower-like self-folding magnetic bilayer hydrogels for artificial-muscle and biomedical applications.^237^ In addition, Tong *et al.*^238^ developed MRHs crawlers that undergo reversible contraction and expansion under alternating magnetic fields and move through confined spaces by exploiting asymmetric friction. In addition, biomimetic magnetic microrobots inspired by magnetotactic bacteria have been fabricated using aligned iron oxide nanoparticle chains embedded in hydrogel microcapsules, enabling magnetically controlled drug transport and release.^239^

#### 5.3.2 Biosensing

Under a magnetic field (MF), magnetic nanoparticles can self-assemble into photonic crystal structures.^187^. Embedding these structures in stimuli-responsive hydrogels enables pronounced color changes in response to environmental variations,^240,241^ supporting biosensing applications. Shang *et al.*^242^ prepared thermochromic photonic fibers by aligning superparamagnetic carbon-coated Fe_3_O_4_ nanocrystal clusters (Fe_3_O_4_@C CNCs) within thermoresponsive poly(*N*-isopropylacrylamide) (PNIPAM) hydrogels before gelation. Upon transferring from air to water, swelling shifted the fiber color from dark green to red; heating to 36 °C reversed the color to green and induced contraction, enabling thermochromic actuation. Similarly, magnetic-field-assisted UV photopolymerization has been used to fabricate photonic hydrogel films containing one-dimensional Fe_3_O_4_@CNC chains. Changes in hydrogel volume alter interparticle spacing and structural color, allowing responses to humidity, ion concentration, and mechanical stress.^243^

Magnetic fields can also directly regulate structural-color hydrogels. Wang *et al.*^232^ incorporated superparamagnetic Fe_3_O_4_@PSSMA@SiO_2_ nanoparticles into acrylamide/polyethylene glycol diacrylate hydrogels (Fig. 16b). Magnetic self-assembly combined with mask-assisted UV polymerization produced tunable photonic patterns whose structural colors changed reversibly during water-induced swelling and light-induced contraction. In addition, ferromagnetic hydrogel actuators containing oriented Co nanorods with high magnetic anisotropy can be programmed by controlling particle orientation and distribution, enabling biomimetic actuation and soft-robotic functions.^244^

### 5.4 Other fields

Beyond biomedical, environmental, and robotic applications, MRHs are also being explored in coatings, temporary sealing, subsurface engineering, and other industrial systems. In these applications, hydrogel-network architecture and magnetic-particle distribution can be tailored to regulate magnetic responsiveness, mechanical behavior, adsorption, and recoverability.

#### 5.4.1 Novel coatings

Iron oxide pigments are widely used inorganic materials in inks, coatings, plastics, and construction products. Polyacrylamide–organosilicon copolymers prepared by copolymerizing acrylamide with octamethyltetrasiloxane can combine polysiloxane and polyacrylamide characteristics. When incorporated into silicone coatings, these copolymers improve antifouling performance without substantially altering the low surface energy, and their inhibition of diatom adhesion increases with acrylamide content.^245^

In addition, Katarzyna *et al.*^139^ developed an MRH with a rough, highly developed surface and strong adsorption capability, indicating potential for coating and dressing applications. Luo *et al.*^234^ prepared a magnetic rheological gel (MRG) based on carbonyl iron particle@polyacrylamide (CIP@PAM) composites dispersed in a water-soluble PAM matrix for use as a temporary plugging agent in unconventional oil and gas reservoir fracturing. As shown in Fig. 16e, PAM was uniformly coated on the CIP surface, improving particle oxidation resistance. Compared with conventional temporary plugging agents, the MRG exhibited strong magnetic responsiveness, controllability, degradability, and recyclability, supporting its application in hydraulic fracturing.

## 6 Conclusion and outlook

### 6.1 Conclusion

This review comprehensively discusses the preparation methods, properties, and applications of MRHs, and draws the following conclusions:

(1) The distinction between soft- and hard-magnetic MRHs should be based on magnetic hysteresis behavior rather than magnetic-particle composition alone. Particle size, crystallinity, domain state, concentration, and surface chemistry can substantially alter magnetic response, and therefore nominally similar magnetic materials may display different behaviors in different MRH systems.

(2) Blending, *in situ* precipitation, and covalent bonding involve distinct trade-offs. Blending provides processing simplicity and scalability but is relatively sensitive to nanoparticle aggregation and leakage. *In situ* precipitation improves particle confinement and distribution but couples nanoparticle formation to ion diffusion, crosslinking density, and reaction pH. Covalent incorporation provides stronger particle–polymer interfaces and improved retention but increases processing complexity. Consequently, fabrication methods should be selected according to application-specific requirements rather than ranked by a single performance parameter.

(3) Surface modification and hydrogel-network design jointly regulate MRH performance. Particle functionalization influences dispersion, interfacial adhesion, leakage, biological interactions, and additional functionalities, whereas network architecture controls swelling, mechanical stability, particle mobility, and actuation. These coupled structure–property relationships explain why numerical properties reported for different MRHs cannot be generalized without considering composition and testing conditions.

(4) MRHs have demonstrated broad potential in drug delivery, magnetic hyperthermia, tissue engineering, wound healing, environmental remediation, soft actuation, sensing, and other intelligent systems. However, clinical and industrial translation will require improved control of nanoparticle retention, fatigue resistance, reproducibility, biological safety, magnetic-field exposure, sterilization, and scalable manufacturing.

### 6.2 Outlook

Despite substantial progress in MRH design and multifunctional integration, a considerable gap remains between laboratory proof-of-concept systems and clinically or industrially deployable materials. Future development should therefore focus not only on adding functions but also on improving reliability, reproducibility, safety, and manufacturability. Key priorities are summarized below:

(1) Nanoparticle leakage remains a fundamental challenge, particularly in physically incorporated systems. Repeated swelling, compression, stretching, and magnetic actuation may progressively damage the network and accelerate particle release. Future studies should evaluate cyclic fatigue, particle retention, magnetic-property retention, and mechanical degradation over application-relevant timescales. Dynamic covalent bonds, reversible coordination interactions, hydrogen-bond networks, double-network structures, and covalent particle anchoring may provide routes toward simultaneously improving toughness, fatigue resistance, and nanoparticle retention.

(2) Small variations in nanoparticle size, surface chemistry, loading, dispersion, crosslinking density, hydration state, or curing conditions can produce substantial differences in magnetic, mechanical, and swelling behavior. Standardized reporting of particle characteristics, magnetization normalization, mechanical-testing conditions, swelling media, and magnetic-field parameters is therefore required. Reproducible structure–property relationships will be particularly important for scale-up and interlaboratory comparison.

(3) Magnetic-field strength alone is insufficient to characterize magnetic stimulation of MRHs. Field gradient, frequency, exposure duration, duty cycle, source-to-sample distance, and local temperature should also be reported. Magnetothermal applications require control of heat generation and tissue-level temperature distributions, whereas magnetomechanical systems should consider repetitive mechanical stimulation and field-induced stresses. Standardized magnetic-field dosimetry would improve comparison among studies.

(4) Biomedical MRHs should be evaluated not only for short-term cytotoxicity but also for inflammation, hemocompatibility, degradation products, nanoparticle and metal-ion release, biodistribution, organ accumulation, and clearance. Sterilization methods must be optimized because irradiation, heat, or chemical sterilants can alter crosslinking, swelling, particle-surface chemistry, and mechanical properties. Because MRHs combine polymeric biomaterials, magnetic components, and external-field devices, translation requires integrated consideration of both material- and device-related risks.

(5) Laboratory fabrication methods must be adapted to reproducible large-scale manufacturing while maintaining particle distribution, magnetic properties, and mechanical performance. Blending may be attractive for high-throughput processing, whereas *in situ* and covalent strategies offer stronger particle confinement but require tighter reaction and purification control. Additive manufacturing and magnetic-field-assisted fabrication may provide routes toward spatially programmed MRHs if printing accuracy, curing, and batch consistency can be maintained.

(6) Once these translational barriers are addressed, MRHs may evolve from externally controlled multifunctional materials toward adaptive systems. Promising directions include injectable and self-healing MRHs formed through dynamic bonding, magnetically programmed anisotropic structures, closed-loop platforms integrating sensing, imaging, drug delivery, and therapeutic feedback, and hybrid systems coupled with piezoelectric, triboelectric, or thermoelectric components for self-powered sensing and stimulation. The key challenge is to integrate these functions without sacrificing mechanical reliability, biological safety, or manufacturability.

## Author contributions

M. Zhang: conceptualization, methodology, software, validation, formal analysis, investigation, resources, data curation, writing – original draft preparation, writing – review & editing, visualization. X. Cui: conceptualization, methodology, software, validation, investigation, resources, writing – review & editing, supervision, project administration, funding acquisition. Y. Zhang: conceptualization, methodology, software, validation, resources, writing – review & editing, supervision, funding acquisition. J. Zhang: software, visualization. Z. Chen: software, visualization. X. Chang: formal analysis. X. Zhao: investigation. M. Liu: validation, data curation. C. Li: resources. All authors read and approved the final manuscript.

## Conflicts of interest

There are no conflicts to declare.

## Data Availability

No primary research results, software or code have been included and no new data were generated or analysed as part of this review.
